# Method for Quantification of Fatty Acids in Ice Cores and Sea-Ice Cores Using Liquid Chromatography High-Resolution Mass Spectrometry

**DOI:** 10.1021/acsmeasuresciau.4c00054

**Published:** 2024-12-13

**Authors:** Siobhán Johnson, Roseanne Smith, Elizabeth Thomas, Chiara Giorio

**Affiliations:** † Yusuf Hamied Department of Chemistry, 2152 University of Cambridge, Lensfield Road, Cambridge CB2 1EW, United Kingdom; ‡ British Antarctic Survey, High Cross, Madingley Road, Cambridge CB3 0ET, United Kingdom

**Keywords:** ice core, sea ice, organic aerosols, biomarker, fatty acids, liquid chromatography mass spectrometry, paleoclimate

## Abstract

Marine-sourced fatty acids provide a promising new suite of proxies for past sea-ice reconstructions, validated using ice cores from Bouvet Island, Greenland, and Alaska. Despite showing great potential as a sea-ice proxy, the transport, deposition, and preservation of these fatty acids within the ice sheet are poorly understood. Additionally, complementary data of the same suite of fatty acids in the source, the surrounding sea ice, is lacking in number, spatial distribution, and seasonal variety, especially in the Antarctic. This study presents an improved method using high-performance liquid chromatography high-resolution mass spectrometry (HPLC-HRMS) for the determination of marine-sourced fatty acids in ice cores and sea ice. The method presents a new preconcentration step using stir bar sorptive extraction (SBSE) as well as reduced background contamination using a trapping column tandem analytical system in HPLC. The method is suitable to detect and quantify a suite of 10 fatty acids with recoveries above 70% and with limits of detection in the low ppb and subppb levels. A range of fatty acids were detected and quantified in samples from two sub-Antarctic ice cores, taken from Peter first Island and Young Island. The results from these cores displayed a variety of fatty acids present in both ice cores (lauric acid, myristic acid, oleic acid, linoleic acid, palmitoleic acid, heptadecanoic acid, pentadecanoic acid, docosahexaenoic acid, eicosapentaenoic acid, and arachidonic acid) as well as a large difference in concentrations between different fatty acids and between the two ice cores. Additionally, this study presents the first results of fatty acid concentrations in the pancake sea ice collected from the Antarctic Marginal Ice Zone.

## Graphical abstract

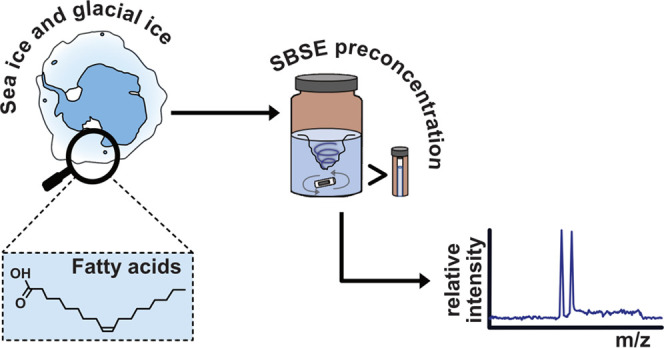

## 1 Introduction

Climate reconstructions, specifically reconstructions of the sea-ice extent around the Antarctic continent, are built on the analysis and quantification of proxy compounds in ice cores. Biogenic marine organic compounds have been detected in continental ice cores from both poles^1–4^ and have shown to be a promising suite of new proxies for sea-ice reconstruction.^5^ In addition to the most widely used methanesulfonic acid (MSA),^6^ low-molecular-weight fatty acids (LFA) sourced from marine phytoplankton can become aerosolized, transported atmospherically, and deposited on ice sheets,^2,7^ either in their primary form or, particularly in the case of the more labile unsaturated fatty acids, as secondary organic aerosols.

There are limited reports on the detection and quantification of fatty acids in Antarctic sea ice, which can be used to validate the relationship between ice cores and sea-ice reconstructions. Nichols et al.^8^ in 1989 conducted the first study to investigate the lipid composition of Antarctic sea ice in McMurdo Sound at three sites. However, despite sampling 1.6–2.5 m of sea ice, only the bottom 20 cm was used for fatty acid analysis. Similarly, Nichols et al.^9^ in 1993 reported fatty acid composition of sea ice again and also only sampled the bottom 20 cm of the sea ice collected (Table 1). Both studies detected and quantified fatty acids with gas chromatography–mass spectrometry (GC-MS). Fahl and Kattner^10^ also presented fatty acid concentrations in sea ice from the Weddell Sea in 1993 (Table 1); however, only one sea-ice core was analyzed together with chunks of brown brash ice and platelet ice. They also used GC-MS techniques for the analysis of their samples. These studies and their limited data sets highlight the lack of data on these marine biomarkers produced by the phytoplanktons that reside in the sea ice.

**Table 1:** Summary of Marine-Associated Fatty Acids Investigated in This Study, Their Neutral Formulas, Their Reported Concentration Range, and the Studies in Which They Were Detected in Ice Cores and Sea Ice^a^

compound name	neutral formula	reported concentration range in Antarctic ice cores (ng/g ice)	reported concentration range in Antarctic sea ice (μg/L meltwater)
lauric acid	$C_{12}H_{24}O_{2}$	4.82 ^3,4^	
myristic acid	$C_{14}H_{28}O_{2}$	15.3 ^2–4^	83.3–369 ^9,10^
pentadecanoic acid	$C_{15}H_{30}O_{2}$	3.56 ^3,4^	36.4 ^9,10^
palmitic acid	$C_{16}H_{32}O_{2}$	20.3 ^2–4^	135–93.6 ^8–10^
palmitoleic acid	$C_{16}H_{30}O_{2}$	^2,4^	148–166 ^8–10^
heptadecanoic acid	$C_{17}H_{34}O_{2}$	5.29 ^3,4^	
stearic acid	$C_{18}H_{36}O_{2}$	10.7 ^3,4^	11.2–46.8 ^9,10^
oleic acid	$C_{18}H_{34}O_{2}$	2.4–189 ^1–4^	138–603 ^9,10^
linoleic acid	$C_{18}H_{32}O_{2}$	^2,4^	21.0–187 ^9,10^
nonadecanoic acid	$C_{19}H_{38}O_{2}$		
arachidic acid	$C_{20}H_{40}O_{2}$	2.03 ^2–4^	
arachidonic acid	$C_{20}H_{32}O_{2}$	^1^	^9^
eicosapentaenoic acid	$C_{20}H_{30}O_{2}$		78.4–582 ^8–10^
heneicosanoic acid	$C_{21}H_{42}O_{2}$		
behenic acid	$C_{22}H_{44}O_{2}$	1.72 ^3,4^	
erucic acid	$C_{22}H_{42}O_{2}$	^2^	
docosahexaenoic acid	$C_{22}H_{32}O_{2}$		18.9–130 ^8–10^
tricosanoic acid	$C_{23}H_{46}O_{2}$	^4^	

a Where there is no reported concentration range given, the reference provided reports either relative proportions of the fatty acids (not absolute concentrations) or the study detected the fatty acid, but it was found below the limit of quantification. Where a range is not reported in the study, the reported average concentration is given.

A small number of studies have detected a range of fatty acids in ice cores and show their potential as sea-ice biomarkers. Kawamura et al.^2^ found total fatty acids at concentrations between 1.9 and 105 ng/g ice (average 20 ng/g ice) throughout a 450-year Greenland ice core, using extraction and esterification followed by analysis with GC-MS. Pokhrel et al.^3^ found a predominance (range 0–189 ng/g ice) of even-numbered carbon chain species palmitic (C16:0), myristic (C14:0), and oleic (C18:1ω9) acids, in an Alaskan ice core dating back to 1734 AD, via butyl ester derivatization followed by GC-MS (limit of detection (LOD) of 0.001 ng/g ice, accounting for preconcentration, while percentage recovery was not reported). Both studies attributed a marine biogenic source to these compounds.

For the Antarctic region, Nishikiori et al.^4^ found the same fatty acid species, using esterification and GC-MS, in inland continental core H15, but at much lower concentrations (0.001–4.11 ng/g ice). More recently, King et al.^1^ detected several fatty acid species in a shallow firn core from sub-Antarctic Bouvet Island, but only oleic acid (C18:1ω9) was continuously present above detection limits throughout the core. King et al.^11^ detailed a method for detecting secondary organic aerosol (SOA) components and fatty acids in ice cores using high-performance liquid chromatography with high-resolution mass spectrometry (HPLC-HRMS). The instrument used by King et al.^1^ is different from other typical fatty acid studies wherein they use a GC-MS. The LTQ Velos Orbitrap used by King et al.^1,11^ had a high mass accuracy of <2 mg/L meltwater and a high sensitivity to the target LFAs. Additionally, by working in liquid chromatography, it does not require any solvent switch or derivatization step prior to the analysis, allowing quantification of fatty acids through direct injection.^12^

As fatty acids are often found in very low concentrations in continental ice samples (parts per billion (ppb) or lower), compared to sea-ice samples (close to parts per million (ppm) and lower),^1–3,13^ many of the methods described incorporate a sample preconcentration step to bring the target analytes above detection limits. King et al.^11^ described three methods of preconcentration: stir bar sorptive extraction (SBSE), rotary evaporation, and solid-phase extraction (SPE).

Rotary evaporation has been used previously in studies of fatty acids in snow and ice samples as well as for the detection of isoprene and monoterpene secondary organic aerosol tracers in snow and ice.^1–3,14^ Studies that have used rotary evaporation to preconcentrate their samples evaporate the liquid meltwater leaving the residual target compounds for analysis. Typically, the compounds are eluted again in a smaller volume of solvent, thus increasing the target analyte concentration. This preconcentration technique is suitable for a wide range of compounds, as discussed by King et al.;^11^ however, it is time-consuming and the recovery is dependent on the starting volume of the sample. King et al.^11^ reported an average recovery of 67% for the analyzed fatty acids using rotary evaporation.

SPE is the most widely used preconcentration technique and another one that has previously been used for organic compounds in snow and ice samples.^11,13,15–17^ This process involves passing the liquid sample through a sorbent mass in a cartridge with a series of washes and eluting the sample to remove the nontarget compounds. There are a wide range of available cartridge types and sorbent masses making selection and optimization complex. King et al.’s^11^ is the only study to have investigated this technique with LFAs in an ice core. They tested three cartridges (C18 PerkinElmer cartridge, Phenomenex Strata-X X-A, and Thermo Fisher Scientific HyperSep SAX) and reported recoveries of the target LFAs after using a HyperSep SAX cartridge. Their results showed low recovery of less than 50% for the investigated LFAs.^11^

King et al. found that the SBSE method was proven to be most effective for LFAs, with an average recovery of 60%. SBSE has also been used for preconcentration of snow and ice samples for extraction of glyoxal and methylglyoxal by Müller-Tautges et al.,^13^ with recoveries of 78.9 ± 5.6 and 82.7 ± 7.5% respectively. Similarly, Lacorte et al.^18^ utilized SBSE for a range of trace (pg/g) persistent organic pollutants in Arctic ice and determined recoveries of their target analytes between 71 and 139% (standard deviation 1–25%). This method is not as time-consuming as rotary evaporation or as complex to optimize as solid-phase extraction and has shown good recoveries for LFAs in ice and snow samples. However, further work is needed to expand the results from these studies for LFAs in particular and improve their recoveries.

This study expands the work of King et al.^11^ by optimizing a method of SBSE-based preconcentration and detection and quantification using HPLC-HRMS to determine the concentration of LFAs in ice cores and sea ice. An expanded list of target fatty acids was identified with reference to published studies of snow and continental ice and sea ice^1–3,8–10^ (Table 1). Additionally, these compounds were selected based on their availability of laboratory standards for calibration and quantification. An analytical method has been developed to improve recoveries with SBSE of a larger list of marine-sourced fatty acids, improved background contamination levels, and method detection limits. The optimized method has been applied to samples from two ice cores collected at Peter first Island and Young Island as well as pancake sea ice collected from the Antarctic Marginal Ice Zone.

## 2 Materials and Methods

Preconcentrated samples were analyzed using high-performance liquid chromatography (HPLC) electrospray ionization (ESI) high-resolution mass spectrometry (HRMS) with a postcolumn injection of ammonium hydroxide in methanol.^11,12^

### 2.1 Chemicals and Reagents

Acetonitrile (>99.9%, Optima HPLC/MS, Fisher Chemical) was used for the preparation of bulk standard solutions. Standard solutions of each analyte were prepared at a concentration of 100 ppm in acetonitrile. These standards were lauric acid (97.9%, European Directorate for the Quality of Medicines & HealthCare), myristic acid (≥99.5%, Fluka), pentadecanoic acid (99%, Alfa Aesar), palmitic acid (≥99%, Fluka), palmitoleic acid (≥99%, Cayman Chemical), heptadecanoic acid (≥98%, Sigma-Aldrich), stearic acid (≥98%, Cayman Chemical), oleic acid (>99%, Sigma-Aldrich), linoleic acid (≥98%, Cayman Chemical), nonadecanoic acid (≥99.5%, Fluka), arachidic acid (≥99%, Sigma-Aldrich), arachidonic acid (95%, Sigma-Aldrich), eicosapentaenoic acid (≥98%, Cayman Chemical), heneicosanoic acid (≥98%, Cayman Chemical), behenic acid (99%, Sigma-Aldrich), erucic acid (>99%, Matreya, LLC), docosahexaenoic acid (≥98%, Cayman Chemical), and tricosanoic acid (>99%, Sigma-Aldrich). The standard solutions were then combined into a diluted standard mixture of all analytes at a concentration of 1 ppm in acetonitrile.

Deuterated internal standards were prepared in a standard bulk concentration of 1 ppm in acetonitrile. These internal standards are d31-palmitic acid (99%, Sigma-Aldrich), *d*_23_-lauric acid (≥98%, Sigma-Aldrich), *d*_9_-oleic acid (95%, Broadpharm), *d*_34_-behenic acid (≥99%, Cayman Chemical), and *d*_35_-stearic acid (≥99%, Cayman Chemical). All standards were stored at −18 °C.

Methanol (>99.9%, Optima UHPLC/MS, Fisher Chemical), and Milli-Q ultrapure water (Milli-Q Advantage A10) were used as eluents. Ammonium hydroxide (25% in water, LC-MS grade, Honeywell Fluka) was used as an additive in the eluents.

### 2.2 Cleaning Procedures and Solvent Purification

All glassware was baked in a furnace (Carbolite Gero CWF 1100 Chamber Furnace) at 450 °C for 8 h following the method of Müller-Tautges et al.^13^ The glassware was capped with PTFE-lined septa. Solvents, used as eluents and for the preparation of diluted standard solutions, were ozonated to remove any background unsaturated fatty acids following the ozonolysis method outlined by King et al.^11^ Briefly, synthetic air was directed into a tubing system, part of which was enclosed by a UV lamp (185:254 nm, Appleton Woods) to generate high (∼290 ppm) concentrations of ozone within the air stream. A mass flow controller was used to regulate the air flow rate at 0.2 L/min as it was bubbled directly through a precleaned glass pipet inserted into the jar of solvent. Solvents were ozonated for 1 h per liter. The solvents were then sonicated for 15 min to remove any residual dissolved ozone. Only unsaturated fatty acids are removed through ozonolysis. For saturated fatty acids, the background contamination is shifted at longer retention times using a two-column system for the chromatographic separation (see Section 2.4 for details).

### 2.3 Sample Preparation

Samples were preconcentrated by SBSE using poly­(dimethylsiloxane) (PDMS)-coated stir bars (Gerstel Twister, film thickness 1 mm, length 10 mm). These have been used in previous studies^11,13,18^ to extract organic compounds, such as fatty acids, from a liquid matrix.

For both standards and environmental samples, 10 mL of the liquid sample, previously spiked with the internal standards at a concentration of 5 μg/L water, was stirred at 700 rpm using a PDMS stir bar for 20 h at room temperature (∼18 °C) in a class-100 clean room. The stir bar was then removed using metal tweezers, placed onto a prebaked foil in the dark until visibly dry, and then transferred into an HPLC vial containing 1 mL of methanol with 0.5 mM ammonium hydroxide. After sonication for 15 min to allow desorption of the analytes into the methanol matrix, the stir bars were removed, and the sample was further concentrated by evaporation using a gentle flow of nitrogen. This produced a final volume of 0.5 mL, corresponding to a theoretical preconcentration factor of 20. A schematic of the sample preparation procedure is reported in Figure 1.

**Figure 1:**
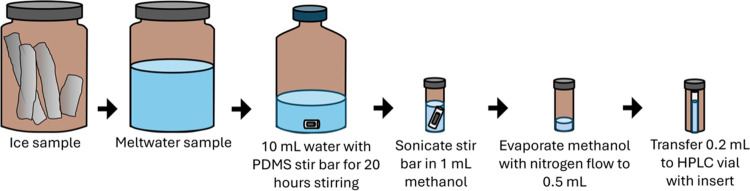
Schematic showing SBSE preconcentration steps from an ice sample. Following melting, the organic fraction is extracted via adsorption onto the PDMS coating of the stir bar. Analytes are subsequently desorbed into a smaller volume of methanol, which is then transferred to a HPLC vial after further concentration from evaporation under nitrogen.

### 2.4 Instrumental Analysis

Samples were analyzed in HPLC-ESI-HRMS using an Accela system HPLC (Thermo Scientific, Bremen, Germany) coupled to an LTQ Velos Orbitrap (Thermo Scientific, Bremen, Germany). Two columns are used in series for chromatographic separation of the analytes: a Waters XBridge C18 (3.5 μm, 3.0 mm × 150 mm) column was used as a trapping column, placed between the eluent mixer and the injection valve, followed by a Kinetex C8 analytical column (2.6 μm, 3.0 mm × 100 mm) (Figure 2). Mobile phases were (A) water with 0.5 mM NH_3_ and (B) methanol with 0.5 mM NH_3_. Separation was done at room temperature (∼20 °C), with a flow rate of 250 μL/min as outlined by King et al.^11^ The elution gradient was: 0–3 min 0% B, 3–4 min linear gradient from 0 to 30% B, 4–9 min 30% B, 9–10 min linear gradient from 30 to 100% B, 10–25 min 100% B, 25–26 min linear gradient from 100 to 0% B, 26–35 min 0% B. In addition, a postcolumn injection of methanol with 5 mM NH_3_ was added at 100 μL/min. The injection volume of each sample was 20 μL. All analytes were quantified in negative ionization using the following ESI source parameters: 400 °C source temperature, 40 arbitrary units (a.u.) sheath gas flow rate, 20 au auxiliary gas flow rate, 3.5 kV needle voltage, 350 °C transfer capillary temperature, and S-Lens RF Level 50% as used in previous studies.^11,12^ MS spectra were collected in full scan, with a nominal resolution of 100,000 at *m*/*z* 400, in the mass range *m*/*z* 80–600. The mass spectrometer was calibrated routinely to within an accuracy of ±2 mg/L using a Pierce LTQ Velos ESI Positive Ion Calibration Solution and a Pierce ESI Negative Ion Calibration Solution (Thermo Scientific, Bremen, Germany). Flow to the LTQ Velos Orbitrap MS was diverted after exiting the two HPLC columns, for the first 8 min of analytical time for all sea-ice samples to prevent any disruption to the MS ion source from the relatively high salt content of the samples. Quantification was done using external calibration for each target fatty acid with standard solutions in the range of 1–200 μg/L in methanol prepared by diluting the 1 mg/L stock standard mixture. The five deuterated internal standards were added to all calibration solutions at a concentration of 100 μg/L. The five internal standards were matched with the 18 target fatty acid species based on their structural similarity (see Section 3.2.2). Calibration was done through linear regression with *x* being the concentration of the analyte over the concentration of the internal standard and *y* being the peak area of the analyte over the peak area of the internal standard, with the internal standard concentration being kept constant. Quality check standards at a concentration of 100 μg/L were also analyzed every 10 samples. No peak broadening was observed with the injection of standard solutions prepared in methanol even if the chromatographic run starts from 0% organic phase.

**Figure 2:**
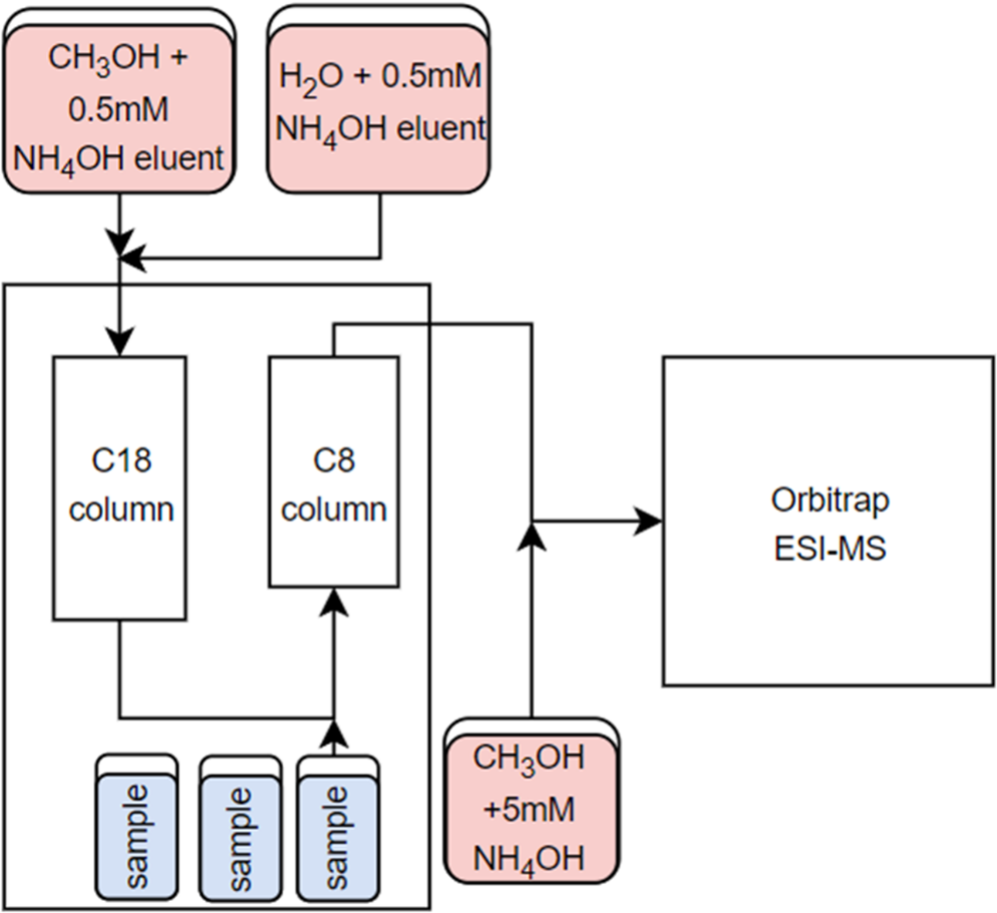
Schematic of HPLC-ESI-MS set up and sample flow.

### 2.5 Method Validation

The instrumental limit of detection (LOD) for each fatty acid was calculated using the Hubaux–Vos method, as recommended by IUPAC.^19,20^ The limit of quantification (LOQ) was calculated as 10/3 × LOD. Instrumental variability was calculated as the relative standard deviation between repeat injections of the same sample from the same vial, while method repeatability was calculated as the relative standard deviation between repeat injections of different samples with varying concentration levels.

Analyte recoveries were determined using standards prepared with 10 mL of ozonated Milli-Q water at a concentration of 5 μg/L for all compounds, including the five internal standards. The effect of starting concentration of the fatty acid was also tested by carrying out a test with samples containing 1 μg/L bulk standard solution of all compounds compared to the 5 μg/L standard.

In order to assess method recoveries for sea-ice samples, standard samples were made up also in salt water (5 g/kg NaCl in milli-Q water) and preconcentrated using the same method with a starting concentration of 5 μg/L of all analytes and internal standards.

The potential for saturation of the stir bars (or column) during (after) preconcentration of environmental samples was assessed by preconcentrating and analyzing a series of standards of increasingly high starting concentration. Standards at starting concentrations of 0, 1, 2, 4, and 7 μg/L were made up to 50 mL using a matrix of ice core meltwater from the Dyer Plateau Antarctic ice core.^21^ This ice is expected to have low background concentrations of organic compounds, due to the core’s high elevation (2000 m above sea level), while enabling the matrix of the standards to more closely replicate true ice core samples. The standards (hereafter referred to as “Dyer preconcentrated standards”) were spiked with a d31-palmitic acid internal standard to give a starting concentration of 1 μg/L and preconcentrated following the method outlined in Section 2.3. This produced a 100× preconcentration factor and final theoretical concentrations of 0, 100, 200, 400, 700 μg/L and 1 mg/L, respectively, assuming full analyte recovery.

The impact of the mode and duration of sample storage was also investigated. First, storage of the preconcentrated samples was considered: 200 μL of the preconcentrated standard was analyzed via HPLC-HRMS immediately following stir bar desorption and evaporation steps (see Section 2.3), while the remaining 300 μL was stored at −18 °C for 1 month prior to analysis, to determine the degree of compound loss (or gain) when the preconcentrated samples are stored at freezer conditions in their methanol matrix prior to analysis.

A second test considered how the storage of firn core sample meltwater affects the preservation of fatty acids in the stage before sample preparation. The method and duration of storage were investigated using a single annual sample of ice from Peter first Island firn core (see Section 2.6). The ice was cut using organic-clean protocols, melted overnight in a dark fridge, and then split into four parts. Part A was transferred directly to an amber glass HPLC vial, spiked with a bulk internal standard (containing five deuterated fatty acid species) to a concentration of 20 μg/L, and placed into the autosampler of the HPLC-HRMS system for same-day analysis (delay between start of melting and analysis of 27 h). Parts B and C were treated identically, except the spiked vials were placed in a dark fridge at 4 °C for several days prior to analysis (delay between the start of melting and analysis of 54 and 151 h for B and C, respectively). Part D was refrozen (after 17 h) at −25 °C for 54 days, remelted, spiked with the internal standard, and analyzed 27 h after the second melt.

### 2.6 Ice Core and Sea-Ice Samples

The optimized method was applied to samples from two firn cores from glaciated sub-Antarctic islands and one sea-ice core from the Weddell Sea. Two firn samples were sourced from the Peter first Island core (Bellingshausen Sea), and an additional sample was obtained from the Young Island firn core (western Ross Sea), both drilled in 2017 using a Kovacs ice corer.^22^ Drilling methods and site details are provided by Thomas et al.^22^

Sea-ice samples analyzed in this study were collected on the SA Agulhas II during the SCALE 2022 Winter Cruise. A sea-ice core was taken from a pancake floe, named OD3, collected from 59° 9′ 42.912″ S to 0° 52′ 22.512″ E on 24 July 2022.^23^

Core locations are shown in Figure 3. All cores were stored in ethylene-vinyl-acetate-treated (EVA) polythene bags at −25 °C in the ice core laboratories at the British Antarctic Survey in Cambridge, U.K.

**Figure 3:**
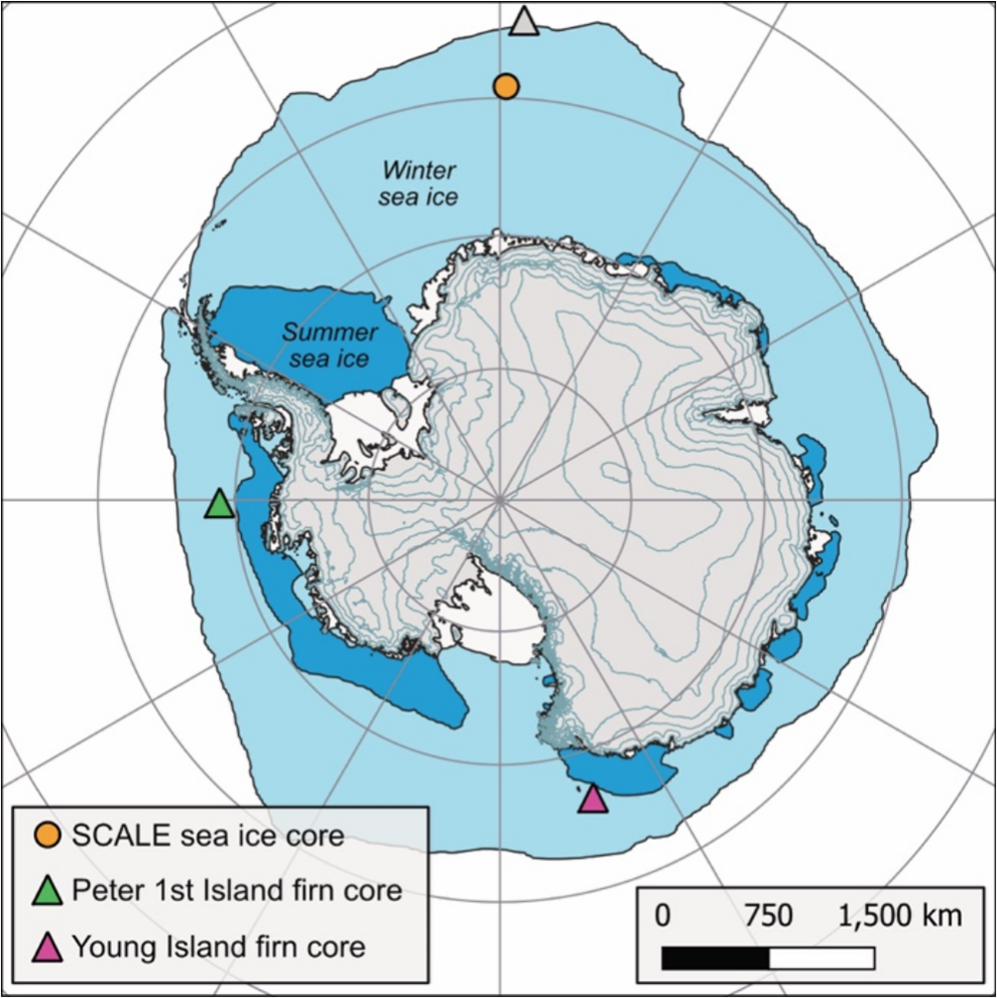
Map showing the location of firn and sea-ice cores used in this study. Colored markers show samples used in this study. An additional gray marker shows the location of Bouvet Island firn core,^1^ referred to in the text.

Samples were cut using a cleaned steel bandsaw blade. Outer sections were removed to reduce contamination, and organic-clean protocols were followed throughout, as per King et al.^11^ The Peter first samples were cut at an annual resolution to provide 2 adjacent years for comparison, with year boundaries set to winter (approximately the end of June) to preserve the summer peak in biogenic species. The Young Island sample was cut at a lower bulked (>1 year) resolution and judged including at least one annual cycle. The sea-ice samples were cut into 5 cm segments after 6 months of storage.

A cleaned ceramic knife was then used to scrape all edges of each piece before transferral to precleaned glass jars with PTFE-lined septa. All samples were melted in the dark at 4 °C and then prepared inside a class-100 clean laboratory. The sea-ice sample meltwater was filtered using 0.4 μm, followed by a 0.2 μm syringe filter before analysis. This was to prevent any large particulate matter from blocking the tubing, capillary lines, or columns during analysis.

## 3 Results and Discussion

### 3.1 Optimization of the Chromatographic Separation

Analyte separation in liquid chromatography was optimized in order to decrease the background contamination of fatty acids naturally present as ubiquitous species in many solvents and surfaces with the aim of improving previously reported detection limits. King et al.^11^ tested a variety of columns, eluents, additives, and eluent gradients, as well as postcolumn additions to improve analyte ionization; the optimized method presented by King et al.^11^ was used as a start point in this study, as it was reportedly optimized for retention times of low-molecular-weight fatty acids.

Using the same instrument as in this study, King et al.^11^ reported LODs ranging between 1.23 and 20.1 μg/L in direct injection (without preconcentration). However, for some fatty acids, e.g., palmitic and stearic acids, the background contamination from the blank chromatographic run was so high that it hindered their quantification. In order to reduce the impact of contamination introduced by the eluents, a combination of two chromatographic columns was used to separate the target fatty acids (Figure 2) in which a C18 trapping column is installed between the eluent mixer and the injection valve, followed by a C8 analytical column mounted after the injection valve. As the retention times were markedly longer (by about 1 min) for the C18 column compared to those for the C8 column, a fatty acid analyte present in the eluent would be shifted at a longer retention time compared to the same analyte present in the actual injected sample. Figure 4 shows an example of an extracted ion chromatogram for palmitoleic acid where two peaks can be clearly identified, one corresponding to the analyte present in the sample and another corresponding to the analyte present as contamination in the eluents. Using a trapping column in which the contamination is eluted at each chromatographic run has the advantage of ensuring a good efficiency of the trapping column, which does not become saturated over time. However, as the contamination is being eluted, it could impact the ionization efficiency of coeluted analytes. The repeatability of the elution is ensured by maintaining a constant elution program and equilibration time (see Section 2.4 for timings of the chromatographic separation and equilibration time at the end of the separation). No coelution of analytes and contamination peaks have been observed. The use of a trapping column increased the sensitivity of the method and improved the LOD for fatty acids (Table 3). For example, our improved method has an LOD of 0.57 μg/L for oleic acid compared to 20.1 μg/L of King et al.^11^ using the same instrument (Table 3). In addition, palmitic and stearic acids are quantifiable with our improved method, albeit with larger LODs compared to other analytes (Table 3).

**Figure 4:**
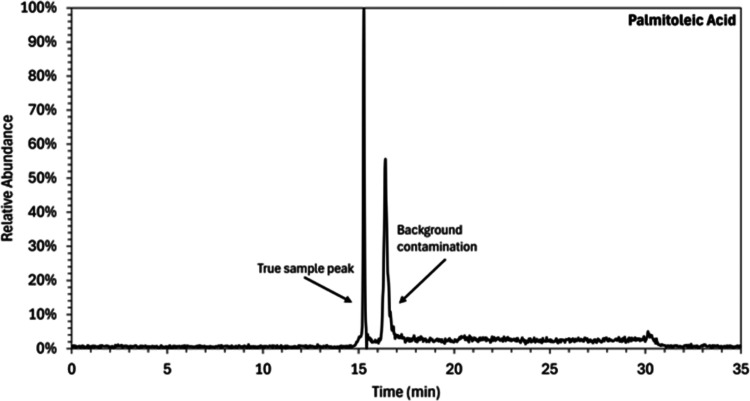
Example extracted ion chromatogram for palmitoleic acid corresponding to the *m*/*z* range of 253.2148–253.2198. The first large peak at RT 15.29 indicates the presence of the fatty acid in the injected sample, while the second peak at RT 16.57 shows the fatty acid that is present as contamination in the eluents.

### 3.2 Optimization of the Preconcentration

#### 3.2.1 Preconcentration of Standard Samples

The PDMS stir bar (GERSTEL Twister) was used for extraction of the fatty acids from water, to then be desorbed into methanol prior to analysis in HPLC-HRMS. The proposed optimized method by King et al.^11^ was carried out with the additional step of evaporating the solvent containing the extracted fatty acids using a gentle flow of pure nitrogen. The resulting 0.5 mL sample was analyzed and the recovery of each compound was quantified.

The standard samples containing 5 μg/L of each of the 18 fatty acids produced final preconcentrated solutions at a theoretical final concentration of 100 μg/L of each fatty acid, assuming 100% recovery. The deviation from this value is used to find the true recovery of each compound (Table 2). The percent recoveries of the fatty acids varied markedly, and some species showed a large variability in their recovery between stir bars (Figure 5). In the following discussion, the compounds are categorized into three groups based on their recovery values.

**Table 2:** Compound-Specific Limit of Detection Achieved Using a Linear Calibration Method, of Standard Solutions with Concentrations Values 1, 10, 50, 100, and 200 μg/L, Listed in the Order of Lowest to Highest Retention Time for the Chromatographic Method^a^

compound name	retention time (min)	LOD (μg/L) [this study]	LOQ (μg/L) [this study]	LOD (μg/L) [King et al.^11^]	LOQ (μg/L) [King et al.^11^]	instrumental repeatability (%RSD)	method repeatability (%RSD)	recovery (%) [this study]	recovery (%) [King et al.^11^]
lauric acid	15.20	3.96	13.2	4.47	14.9	1.97	18.3	91 ± 25	22.0 ± 1.0
myristic acid	15.37	0.55	1.85	19.1	63.8	1.47	3.51	101.0 ± 9.0	65.0 ± 5.0
pentadecanoic acid	15.49	0.44	1.47			1.23	3.53	110.0 ± 6.0	
palmitic acid	15.63	16.7	55.6			3.69	4.00	131 ± 73	
palmitoleic acid	15.46	0.48	1.58			1.50	3.27	109.0 ± 9.0	
heptadecanoic acid	15.76	0.78	2.59	6.27	20.9	1.10	5.95	73 ± 14	62.0 ± 1.0
stearic acid	15.90	30.9	103			6.06	15.6	92 ± 67	
oleic acid	15.72	0.57	1.90	20.1	67.1	1.13	2.50	106.0 ± 4.0	75.0 ± 2.0
linoleic acid	15.57	0.37	1.23			1.22	3.48	113.0 ± 8.0	
nonadecanoic acid	16.05	1.29	4.31	2.00	6.67	1.34	11.8	23.0 ± 3.0	54.0 ± 2.0
arachidic acid	16.24	10.5	35.1			3.66	17.6	18.0 ± 5.0	
arachidonic acid	15.57	0.38	1.26	4.69	15.6	1.72	6.68	113 ± 11	48 ± 16
eicosapentaenoic acid	15.43	0.41	1.38			1.58	6.92	108 ± 13	
heneicosanoic acid	16.43	2.77	9.24			1.51	32.3	6.9 ± 3.0	
behenic acid	16.64	3.51	11.7	5.93	19.8	1.29	63.4	4.4 ± 3.0	38.0 ± 1.0
erucic acid	16.33	1.08	3.59			1.40	11.7	37.0 ± 6.0	
docosahexaenoic acid	15.54	0.38	1.27			1.74	8.17	114 ± 14	
tricosanoic acid	16.88	3.96	13.2	4.47	14.9	1.97	18.3	91 ± 25	22.0 ± 1.0

a Also presented are retention time, limit of quantification, instrument repeatability (i.e., variability between repeat injections of the same sample into the same instrument), method repeatability (variability between different samples prepared using the same method and analyzed on one instrument), and recovery (the percentage of the compound recovered from the analysis compared to that which was present in the original sample before preconcentration, as determined using standards of known input values). RSD values of the method and instrumental repeatability were calculated using a 100 μg/L standard for all fatty acids.

**Figure 5:**
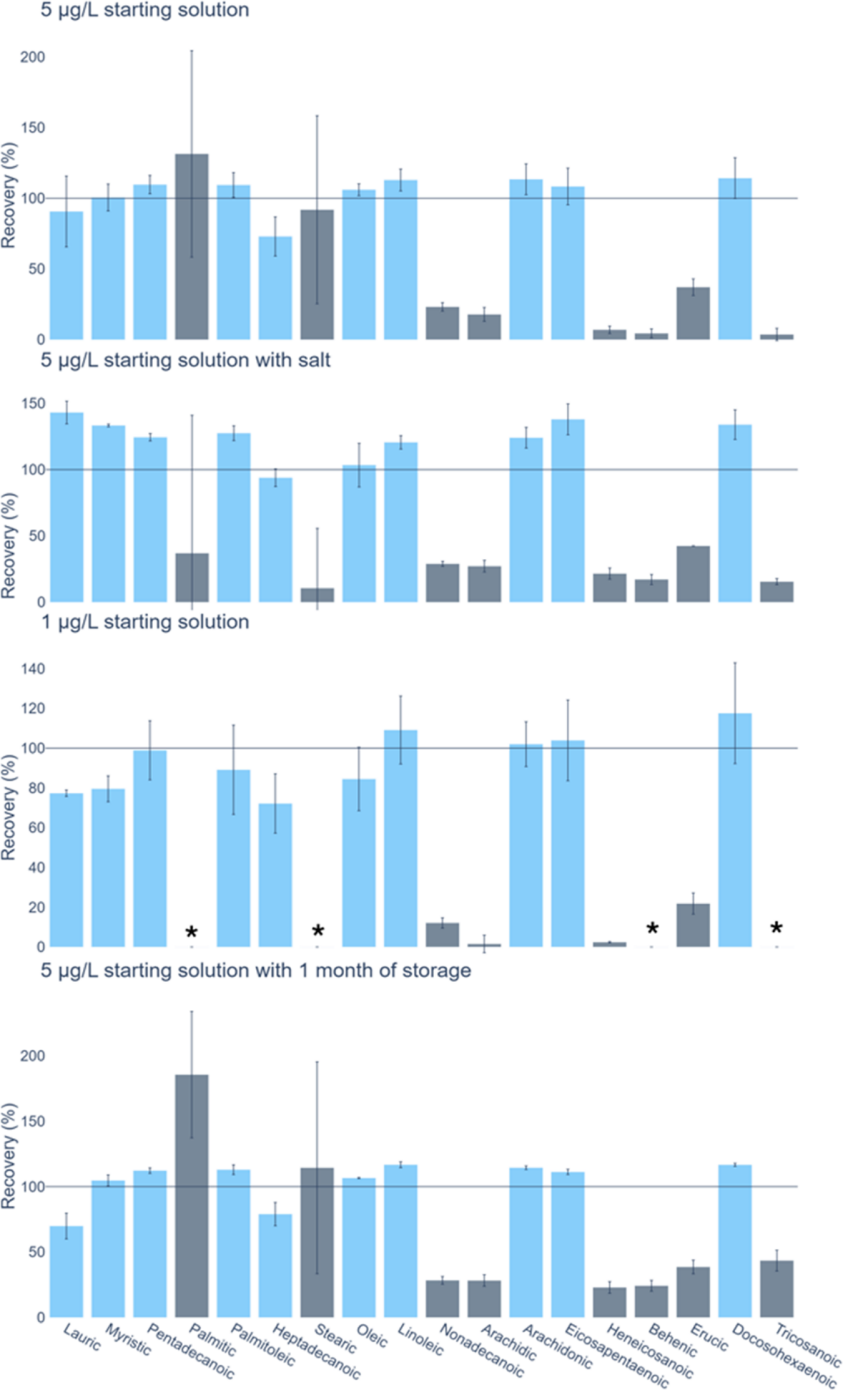
Comparative compound recoveries using SBSE preconcentration for different starting solutions. Horizontal gray line represents 100% recovery, while blue bars are the selected 10 compounds that are found to have an overall good affinity with the stir bars with the optimized method. Asterisks represent compounds that were recovered, but there was too much background contamination for a reliable estimation of recovery and calibration.

The first group includes 10 fatty acids (lauric, myristic, pentadecanoic, palmitoleic, heptadecanoic, oleic, linoleic, arachidonic, eicosapentaenoic, and docosahexaenoic acid). These showed recoveries exceeding 70% and a standard deviation of less than 25%. This group is dominated by a shorter chain and unsaturated species. Several were also targeted by King et al.,^11^ and the recoveries are markedly improved in this study (Table 2). Similar results (>70% recovery, standard deviations < 20%) were achieved for this group of 10 when using a lower starting concentration of 1 ppb (Figure 5). Similarly, the preconcentration test with salt water and a standard concentration of 5 ppb also yielded high recoveries for these 10 fatty acids (Figure 5) (>70% recovery, standard deviations < 25%). The instrumental and method repeatability showed a good performance with coefficient of variations of less than 3 and 6%, respectively (Figure 6). These results demonstrate that this method is suitable for extracting, preconcentrating, and detecting these 10 fatty acids with low variability and high recovery. This method can be used with different starting concentrations and with the inclusion of salts without the detriment to the recoveries of the compounds.

**Figure 6:**
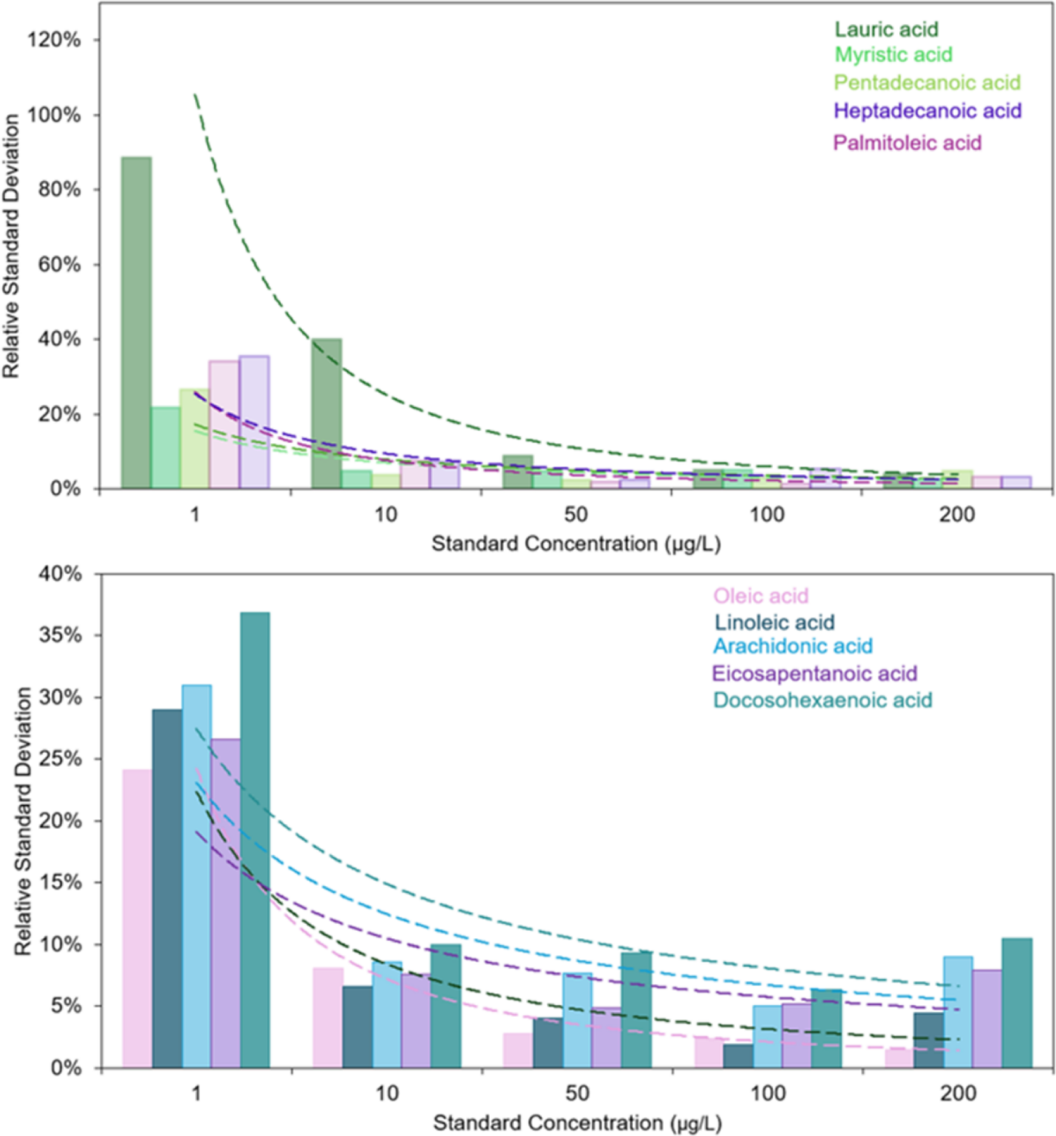
Relative standard deviation of the peak area of 10 of the target fatty acids across the standard levels. The plateaued value of the trend line is the resultant method repeatability for that fatty acid.

A second group of six species, including many of the longer-chain saturated fatty acids and those with odd-numbered carbon chains, were not extracted successfully by the stir bars. Nonadecanoic, arachidic, heneicosanoic, behenic, erucic, and tricosanoic acids had recoveries of below 40% from the 5 μg/L bulk standard solution, with most recovering less than 20% of the available analyte (Table 2). Behenic and tricosanoic acids were the least well-recovered of this group, with recoveries of 0–10% (Figure 5). At the moment, it is unclear why these compounds would have a lower recovery, as their chemical functionalities are analogous to that of the analytes of the first group. The method repeatability (Table 2) was poor for this group, with errors between 11 and 87%. King et al.^11^ investigated the recovery using stir bars for nonadecanoic, behenic, and tricosanoic acids and calculated low recoveries of 54, 38, and 38%, respectively. Despite significant improvement in the recoveries for the aforementioned shorter-chain fatty acids, these did not improve with this study. As a result, preconcentration using PDMS stir bars is considered unsuitable for these compounds.

The third group includes palmitic and stearic acids. These showed high stir bar recovery rates of 131% and 92%, but large standard deviations of 73 and 67%, respectively (Table 2). Both fatty acids are known to be ubiquitous outside the marine environment; thus, it is likely that this large variability results from background contamination. Reliable quantification of the percent recoveries is made difficult by the high rate of background contamination, which also results in high detection limits. For example, 20× preconcentration of the 1 μg/L standard samples would yield final theoretical palmitic and stearic acid concentrations of 20 μg/L, which is below their LOQ. As a result, they could not be reliably recovered at low concentrations (Figure 5). King et al.^11^ chose to exclude palmitic acid from their study because of high contamination. Similarly, this study suggests that this method of extraction, preconcentration, and detection using HPLC-HRMS is unsuitable for both palmitic and stearic acid.

#### 3.2.2 Stir Bar and Column Saturation during Preconcentration

Preconcentration of environmental samples, whose concentration is inherently unknown prior to analysis, has the potential to generate concentrations that exceed the loading capacity of the PDMS stir bars, cause saturation of the chromatographic column, or lead to saturation of the HRMS detector. This may cause, respectively, incomplete stir bar recovery, poor chromatographic separation, or nonlinearity in the instrument response. The potential for such saturation effects was assessed using a series of preconcentrated meltwater standards at increasing starting concentrations, referred to as the ‘Dyer preconcentrated standards’ (see Section 2.5).

The calibration curves produced by the calibration standards (implemented across a similar concentration range, using a methanol matrix) were then compared to the slope of the curves produced by the Dyer preconcentrated standards (also in a methanol matrix following preconcentration) to determine the degree of saturation as the standard levels increase. Figure 7 confirms this effect; apparent reduced sensitivity to the higher Dyer preconcentrated standard levels results in a weaker calibration slope for this data set in comparison to the (nonpreconcentrated) calibration standards, but for varying degrees across target species. These results suggest that incomplete extraction (i.e., reduced recovery) of the analyte by the PDMS stir bars, due to saturation of the PDMS “stationary phase”, may have occurred.

**Figure 7:**
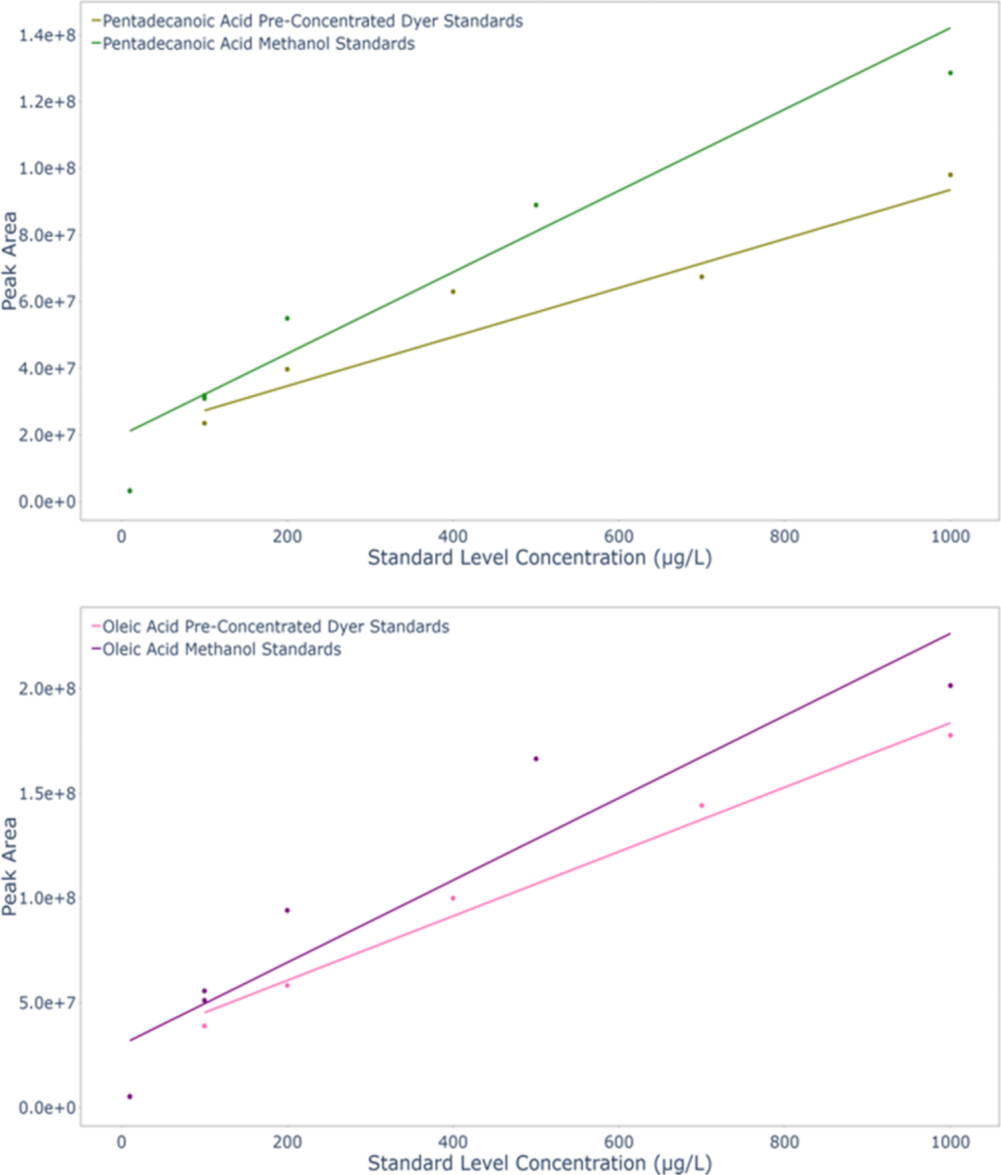
HPLC-HRMS peak area response and fitted linear calibration curves for a series of preconcentrated standards for oleic and pentadecanoic acids at increasing starting concentrations compared to a range of instrumental calibration standards. Shading highlights the difference between the curves, indicating that stir bar saturation has taken place for the higher Dyer preconcentrated standard levels.

Figure 8 displays the instrumental response to the deuterated internal standard. D31-palmitic acid was added to the Dyer standards prior to preconcentration, as well as into all instrumental calibration standards and instrumental blanks. A systematic reduction in the instrumental response is apparent not only for the preconcentrated samples but also for the instrumental calibration standards, which were not subject to preconcentration. This shows that in addition to the reduced stir bar recovery, column saturation has also occurred for the higher preconcentrated standard levels.

**Figure 8:**
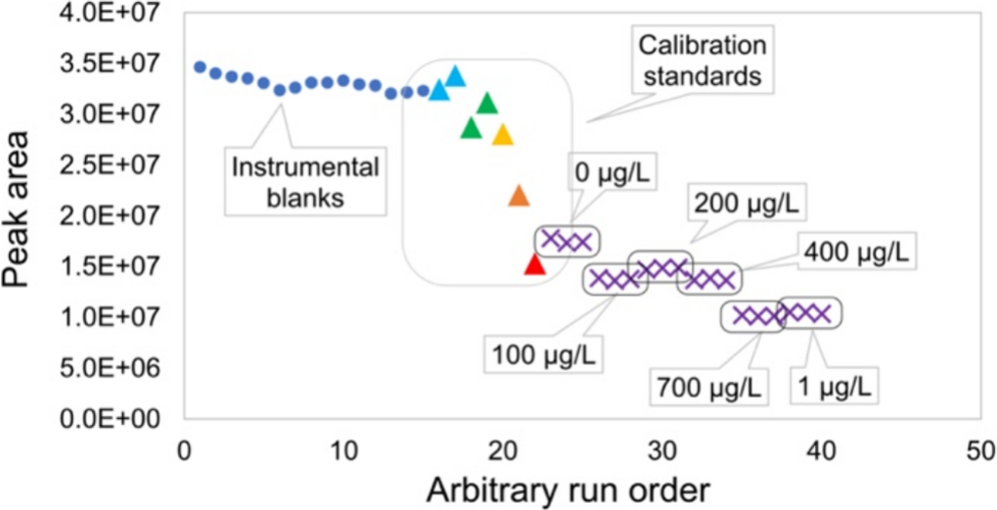
Instrumental response to *d*_31_-palmitic acid across Dyer preconcentrated standards, instrumental calibration standards, and instrumental blanks to assess saturation effects for high-concentration environmental samples. All vials were prepared in a methanol matrix.

To counter these effects, a range of deuterated internal standards are implemented across all samples, standards, and blanks. Any impact of the stir bar, column, or detector saturation upon the target fatty acid compounds can then be corrected through normalization to the peak area response of the internal standard that most closely matches the species’ structure (e.g., chain length, degree of chain unsaturation). Five internal standards were adopted to enable appropriate matching across the suite of 10 fatty acids identified as target species in Section 3.2.1. Lauric acid was corrected using d23-lauric acid, myristic with d23-lauric, pentadecanoic with d31-palmitic, palmitic with d31-palmitic, palmitoleic with d9-oleic, heptadecanoic with d31-palmitic, stearic with d35-stearic, oleic with d9-oleic, linoleic with d9-oleic, nonadecanoic with d35-stearic, arachidic with d35-stearic, arachidonic with d9-oleic, eicosapentaenoic with d9-oleic, heneicosanoic with d43-behenic, erucic with d43-behenic, docosahexaenoic with d43-behenic, and tricosanoic with d43-behenic.

### 3.3 Effect of Storage

Two investigations were carried out to determine how storage of the prepared standards and samples prior to instrumental analysis (i.e., delayed analysis) impacts the concentrations of target fatty acids.

The preconcentrated standards that were stored in freezer conditions for 1 month prior to analysis showed no substantial loss or gain of target compounds when compared to those analyzed immediately; all species (except for arachidic, heneicosanoic, behenic, and tricosanoic acids, which also showed poor recovery by SBSE (see Section 3.2.1)) remained within one standard deviation of the nonstorage concentrations (Figure 5). This suggests that the fatty acids are stable, and samples are viable for analysis, following storage at freezer conditions, in a methanol matrix, for up to a month.

A second test considered how the storage of firn core meltwater influences the preservation of fatty acids in the stage prior to sample preparation. The 2003–2004 annual sample of the Peter first firn core was analyzed immediately via direct injection and six species were detected at concentrations exceeding their limit of quantification: lauric, myristic, palmitic, stearic, oleic, and linoleic. A statistically significant decrease in the concentration of each of these species was observed for the stored parts relative to the part analyzed on the same day (Figure 9). On average, the species were reduced to 87, 74, and 46% of the concentration of part A for parts B, C, and D, respectively. Progressive loss of fatty acids during the time spent in storage may result from microbial degradation,^2^ photodegradation^24^ or other chemical transformation, such as oxidation.^25^ Baked-clean glassware (to minimize bacteria) and dark conditions (reducing light-mediated reactions) are suggested to reduce losses during storage. Fridge storage is preferable to refreezing.

**Figure 9:**
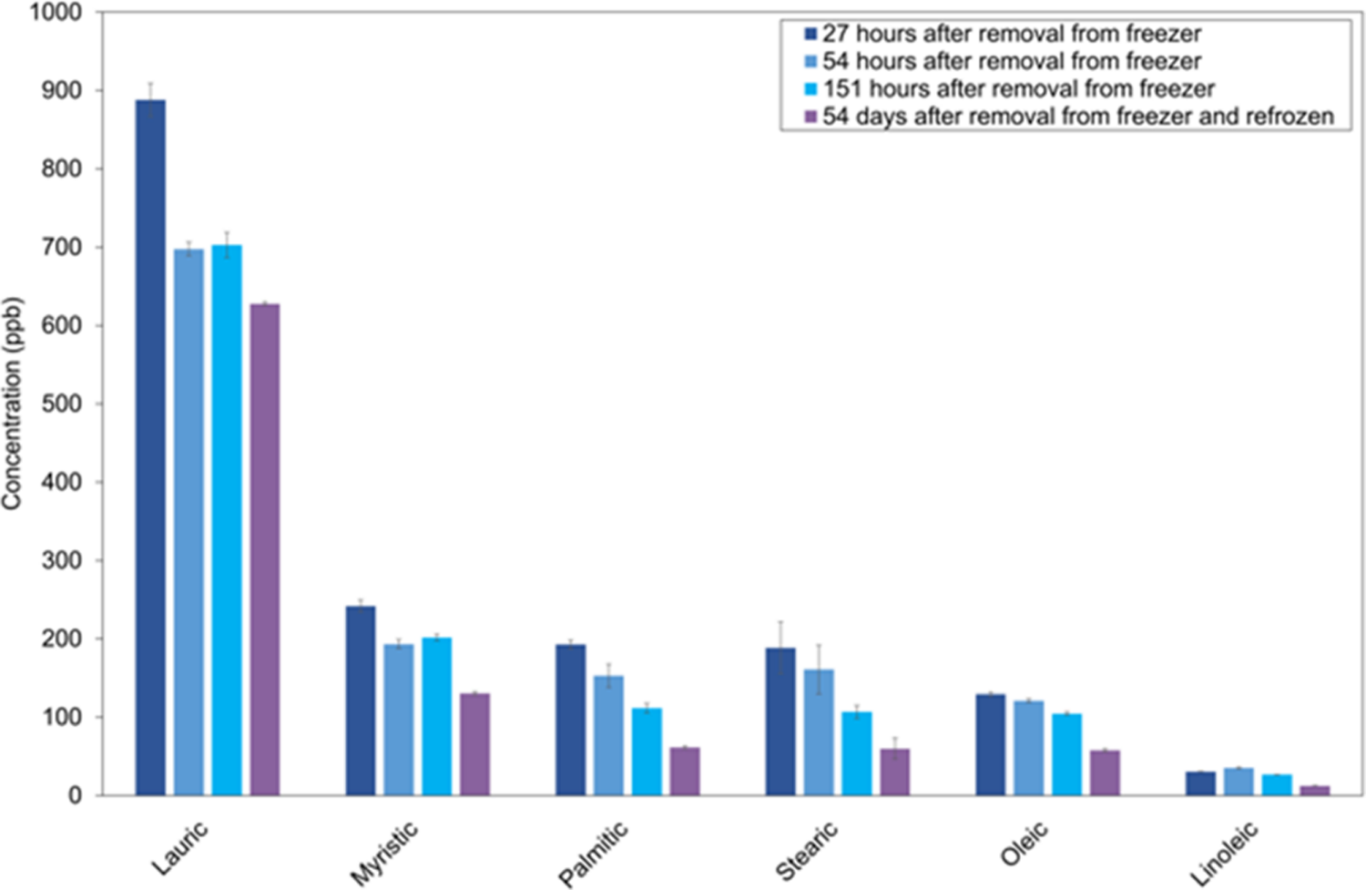
Degradation of fatty acids in ice core meltwater (from the Peter first ice core annual sample 2003–2004) subjected to storage prior to analysis. Error bars show the standard deviation between triplicate injections from the same sample vial. Storage duration counted from the point the sample was transferred from −25 to 4 °C for melt.

### 3.4 Method Application

#### 3.4.1 Peter First Island and Young Island Firn Cores

The full optimized method was applied to two annual samples from sub-Antarctic Peter first Island, and one sample from the Young Island firn core. These samples were preconcentrated, analyzed using the optimized HPLC-HRMS method, and the final fatty acid concentrations were calculated using the respective compound recoveries. For the group of 10 fatty acid species shown to be effectively recovered by the stir-bars (see Section 3.2.1), the calculated concentrations are presented in Table 3. All 10 fatty acids were found in the Peter first samples; six were also found in the Young Island sample.

**Table 3:** Summary of the Results of the SBSE Preconcentration Method Test on Ice Cores and the Final Concentrations of the Selected 10 Fatty Acids in the Peter First Island Ice Core and Young Ice Core^a^

compound name	LOD, this study (μg/L)	LOQ, this study (μg/L)	Young Island ice core (μg/L) [SBSE × 20]	Peter first Island ice core, year 2003–2004 (μg/L) [SBSE × 5]	Peter first Island ice core, year 2003–2004 (μg/L) [direct]	% error between SBSE and direct	Peter first Island ice core, year 2004–2005 (μg/L) [SBSE × 5]	Peter first Island ice core, year 2004–2005 (μg/L) [direct]	% error between SBSE and direct
lauric acid	3.96	13.2	<D/L	906 ± 48	888 ± 21	–2.0	633 ± 23	948 ± 15	33
myristic acid	0.55	1.85	0.12	1561 ± 41	242.0 ± 7.7	–540	1494 ± 40	351.0 ± 4.5	–330
oleic acid	0.57	1.90	0.75	86.00 ± 0.76	130 ± 2.8	34	81.00 ± 0.53	139.0 ± 2.4	42
linoleic acid	0.37	1.23	0.45	27.00 ± 0.36	31.00 ± 0.25	13	24.00 ± 0.46	37.00 ± 0.89	35
pentadecanoic acid	0.44	1.47	0.02	6.80 ± 0.13	1.6	–325	3.50 ± 0.21	1.80 ± 0.32	94
palmitoleic acid	0.48	1.58	0.30	2.000 ± 0.070	2.10 ± 0.25	4.8	2.300 ± 0.090	3.20 ± 0.12	28
heptadecanoic acid	0.78	2.59	0.33	4.10 ± 0.17	<D/L		1.800 ± 0.050	<D/L	
docosahexaenoic acid	0.38	1.27	<D/L	0.028 ± 0.010	<D/L		0.450 ± 0.020	<D/L	
eicosapentaenoic acid	0.41	1.38	<D/L	0.0140 ± 0.0030	<D/L		0.380 ± 0.030	<D/L	
arachidonic acid	0.38	1.26	<D/L	0.0170 ± 0.0040	<D/L		0.300 ± 0.010	<D/L	

a <D/L denotes that the fatty acid was not detected above its limit of detection.

The two samples from the Peter first ice core were analyzed via direct injection alongside the SBSE method, for comparison. Four of the fatty acids (heptadecanoic, docosahexaenoic, arachidonic, and eicosapentaenoic acid) were recovered successfully and detected only following preconcentration; their respective concentrations were too low to be detected via direct injection only.

For the Peter first samples analyzed via direct injection (Table 3), six fatty acid species were found, and the concentrations were similar for both direct injected annual samples. Lauric, myristic, and oleic acids were detected at concentrations between 100 and 1000 μg/L, linoleic acid was found at 30–40 μg/L, while pentadecanoic and palmitoleic acids were detected at lower concentrations nearing their LOQs. Comparing these values to those from the same Peter first samples following treatment with the SBSE preconcentration method, the results are very similar; the concentrations were within one standard deviation of the SBSE recovery. The exception to this was myristic acid, for which a structural isomeric interference partially overlapping the chromatographic peak was observed that may have affected its accurate quantification.

The results from Peter first and Young Island are within a similar order of magnitude to reported values in other ice cores (Table 1). The fatty acid concentrations shown for Peter first are considerably higher than those found for the Young Island sample. This contrasts with a recent study by Segato et al.,^26^ which finds similar concentrations of the marine biogenic-sourced species methanesulfonic acid (MSA) in bulked samples from both Peter first (34 ± 7 ng/g) and Young Island (40 ± 4 ng/g) firn cores. The discrepancy here could owe to the greater degree of melt present in the Young core compared to Peter first.^27^ The Young Island bulked sample used for the analysis in this study, which represented ∼60 cm depth of firn, was selected from ∼8 m depth in the core, from a section shown by Moser et al.^27^ to include some of the largest (>10 cm) melt layers present in the core. It is likely this section suffered from some postdepositional loss of fatty acid species due to elution of organic species by percolating meltwater.

Both sets of samples—Peter first and Young—exceed the reported concentrations in a third sub-Antarctic island firn core, Bouvet Island, which was analyzed by King et al.^1^ using HPLC-HRMS with preconcentration via rotary evaporation. Of their 11 target fatty acid species, only oleic acid was found continuously throughout the Bouvet core. The lower concentrations at Bouvet are unsurprising when considering the location of the islands (see Figure 3); Peter first and Young are both situated close to the phytoplankton source inside the seasonal sea-ice zone, while Bouvet sits at the winter sea-ice edge, northward of the seasonally productive region. Segato et al.^26^ reported average MSA concentrations at Bouvet of just 1.9 ± 0.4 ng/g.

#### 3.4.2 Sea-Ice Cores

Discrete segments along the length of the sea-ice core were analyzed on using the HPLC-HRMS via direct injection to find the concentration profiles of the target fatty acids. The results showed that five fatty acids were detected above their respective LODs, but below their LOQs: lauric acid, myristic acid, pentadecanoic acid, palmitoleic acid, and linoleic acid.

To confidently quantify these fatty acids within the sample and possibly identify more present, the discrete samples were analyzed again after preparation using the stir-bar preconcentration method outlined in Section 2.3. The samples were preconcentrated with a factor of 5 and analyzed on HPLC-HRMS using the same instrumental method.

Using the SBSE preconcentration method, seven fatty acids were detected and quantified above their LOQs. Eicosapentaenoic and oleic acids were successfully recovered and detected on top of the remaining five detected using direct injection.

The concentration profiles of these fatty acids, from the top to the bottom of the sea-ice core, can be seen in Figure 10. The concentrations range from below the detection limit to over 8 μg/L. The median concentrations of each fatty acid in the sea-ice core are 2.24 μg/L for lauric acid, 2.18 μg/L for myristic acid, 0.73 μg/L for pentadecanoic acid, 0.86 μg/L for palmitoleic acid, 0.35 μg/L for oleic acid, 6.07 μg/L for linoleic acid, and 0.58 μg/L for eicosapentaenoic acid.

**Figure 10:**
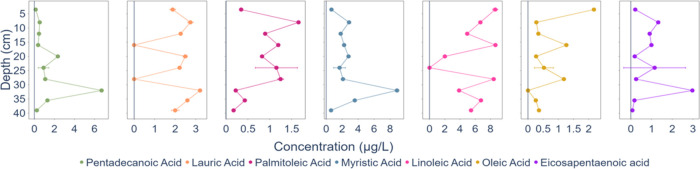
Vertical concentration profiles of detected fatty acids in the pancake sea-ice core ‘OD3’ from the SCALE 2022 Winter Cruise after 5x SBSE preconcentration.

In comparison to reported literature concentrations seen in Table 1, these results show a low fatty acid content by several orders of magnitude. However, the sparsity of data published with regard to fatty acids in Antarctic sea ice^8–10^ means that the full extent of the concentration range of fatty acids is still unknown. Additionally, this sea-ice core was collected during the austral winter, in which the sea ice is still forming, and biological productivity is reported to be low, compared to spring and summer months.^28^ The core was collected from a pancake ice floe, which is a type of sea ice that forms during the first stages of its development before consolidating into larger packs of sea ice.^29^ The pancake floe is thus estimated to have only formed a few days prior to collection, thus limiting the time for microorganisms, such as diatoms and other phytoplankton species, to build up a substantial community within the sea ice.

## 4 Conclusions

This study presents an optimized method of detecting and quantifying LFAs of biogenic marine origin in ice cores and sea ice. The method utilizes SBSE as a means of preconcentrating liquid samples before analysis using HPLC-HRMS. The method is shown to deliver repeatable results for environmental samples in the ppb and subppb ranges, for use in environmental and paleoclimate research.

The study builds on previous work by King et al.,^11^ which targeted a wider range of organic compounds in ice but a smaller range of LFAs. First, steps were introduced to reduce background contamination of fatty acids throughout the method, such as the addition of a second trapping column between the mixer and the injection valve in the HPLC system. The study also employs a preconcentration method using SBSE that is specifically targeted toward LFAs. This study investigated the detection and quantification limits for 18 fatty acids, of which 10 were successfully recovered using the SBSE preconcentration technique with median recoveries of 109% for standard samples and 126% for salt-water standard samples. The effect of starting concentration was investigated; the method worked effectively for starting concentrations as low as 1 μg/L, while at higher concentrations the study showed that unwanted saturation effects (of both stir bars and HPLC column) can be introduced. Thus, a series of internal standards are utilized in the final optimized method, to counter any saturation effects. Therefore, the method is suggested for use in samples ranging from LODs to about 1000 μg/L (after preconcentration).

A secondary investigation into the preservation of the target analytes during sample storage (i.e., delayed analysis) was conducted. It was shown that refrigerating melted samples prior to the preconcentration treatment leads to a gradual decrease in the measured concentration (via degradation), but refreezing the samples results in a greater degree of compound loss. Preferable to both of these options is storing already-preconcentrated samples, in the methanol matrix, at freezer conditions, where sample concentrations were shown to be stable for up to 1 month prior to eventual analysis via HPLC-HRMS.

The full optimized method was tested on two ice (firn) cores and one sea-ice core from Antarctica and was found to successfully identify and quantify a number of fatty acids in both sample types. A comparison between the results from direct injected samples and replicate samples that were treated with the optimized preconcentration method showed the results to be comparable except for one analyte for which a structural isomeric interference may have been present in the tested sample. Thus, this preconcentration technique is an effective route to overcoming low detection limits without an excessive loss of analytes throughout. Moreover, four fatty acids were detectable only after preconcentration was applied, which suggests that new and understudied compounds can be explored in environmental samples using the optimized preconcentration method.

This study is presented to assist in the development of marine-sourced fatty acids as biomarker proxies in the polar regions. These compounds have been suggested to contain important information about past climatic and ecological conditions in the Southern Ocean when applied in paleoclimate research.

## Supplementary Material



## Notes

Additional figures depicting example extracted ion chromatograms (PDF)
