# Supplementary material for: Method for Quantification of Fatty Acids in Ice Cores and Sea-Ice Cores Using Liquid Chromatography High-Resolution Mass Spectrometry
Source: ACS Meas Sci Au. 2024 Dec 13;5(3):264–76. doi: 10.1021/acsmeasuresciau.4c00054 (PMC12183598; doi:10.1021/acsmeasuresciau.4c00054)
Supplement: Supplementary file 1 [file tg4c00054_si_001.pdf]

Supporting Information for

A method for quantification of fatty acids in ice cores and sea-ice cores using liquid chromatography high-resolution mass spectrometry

Siobhán Johnson[a,b*‡], Roseanne Smith[a,b*‡], Elizabeth Thomas[b], Chiara Giorio[a*]

[a] *Yusuf Hamied Department of Chemistry, University of Cambridge, Lensfield Road, Cambridge CB2 1EW, United Kingdom*

[b] *British Antarctic Survey, High Cross, Madingley Road, Cambridge CB3 0ET, United Kingdom*

‡These two authors contributed equally to this work.

*Correspondence to: saj59@cam.ac.uk; rms220@cam.ac.uk; cg525@cam.ac.uk

Table of Contents

16 Figures

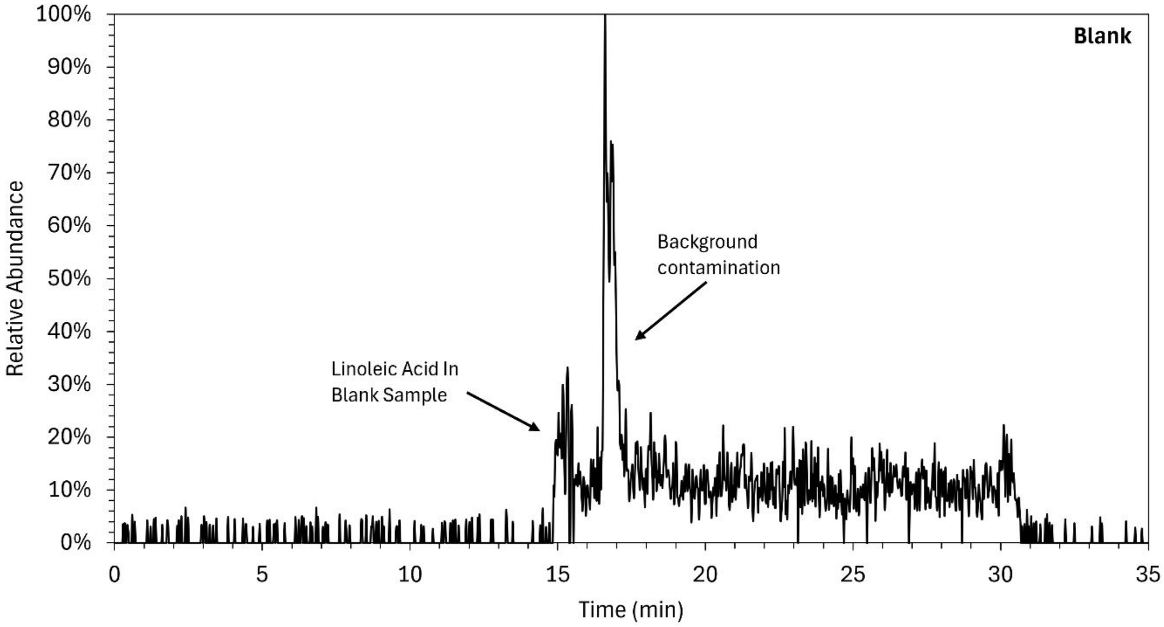

Figure S.1: Example extracted ion chromatogram for linoleic acid in a blank sample corresponding to the m/z range of 279.2301-279.2357. The first large peak at RT 15.34 indicates the presence of the fatty acid in the injected blank sample, which is sample contamination, whilst the second peak at RT 16.61 shows the fatty acid that is present as contamination in the eluents.

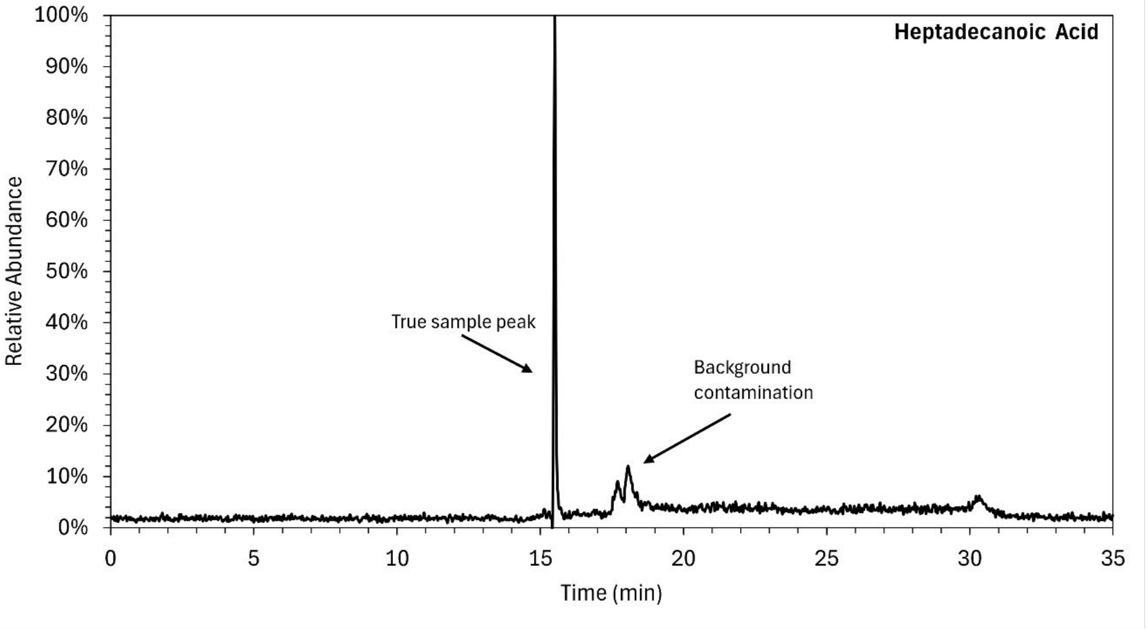

Figure S.2: Example extracted ion chromatogram for heptadecanoic acid in a standard sample corresponding to the m/z range of 279.2301-279.2357. The first large peak at RT 15.34 indicates the presence of the fatty acid in the injected sample, whilst the second peak at RT 16.61 shows the fatty acid that is present as contamination in the eluents.

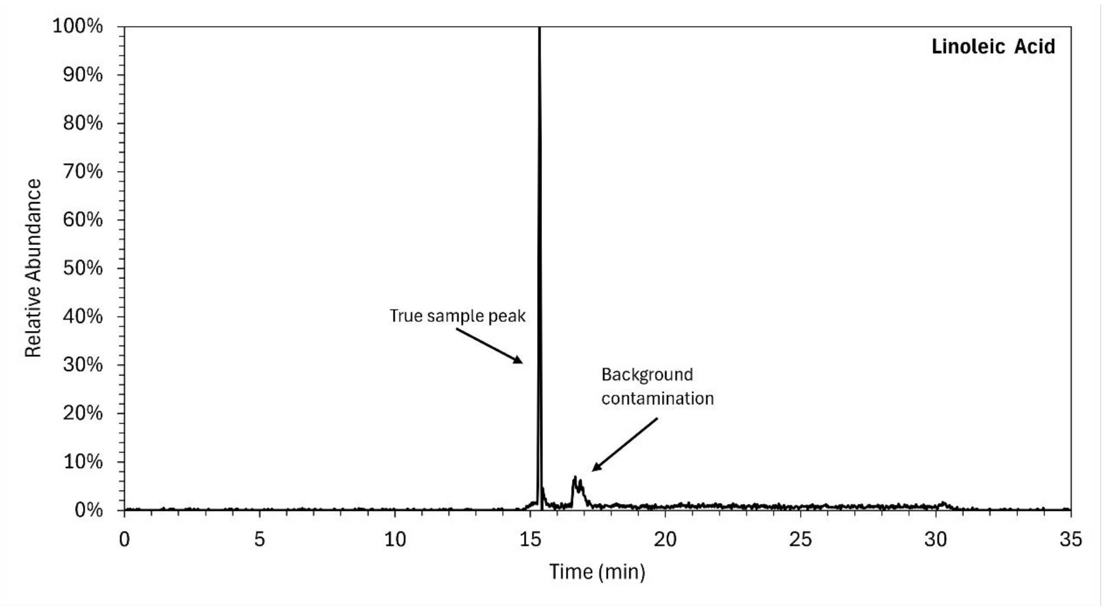

Figure S.7: Example extracted ion chromatogram for linoleic acid in a standard sample corresponding to the m/z range of 279.2301-279.2357. The first large peak at RT 15.34 indicates the presence of the fatty acid in the injected sample, whilst the second peak at RT 16.66 shows the fatty acid that is present as contamination in the eluents.

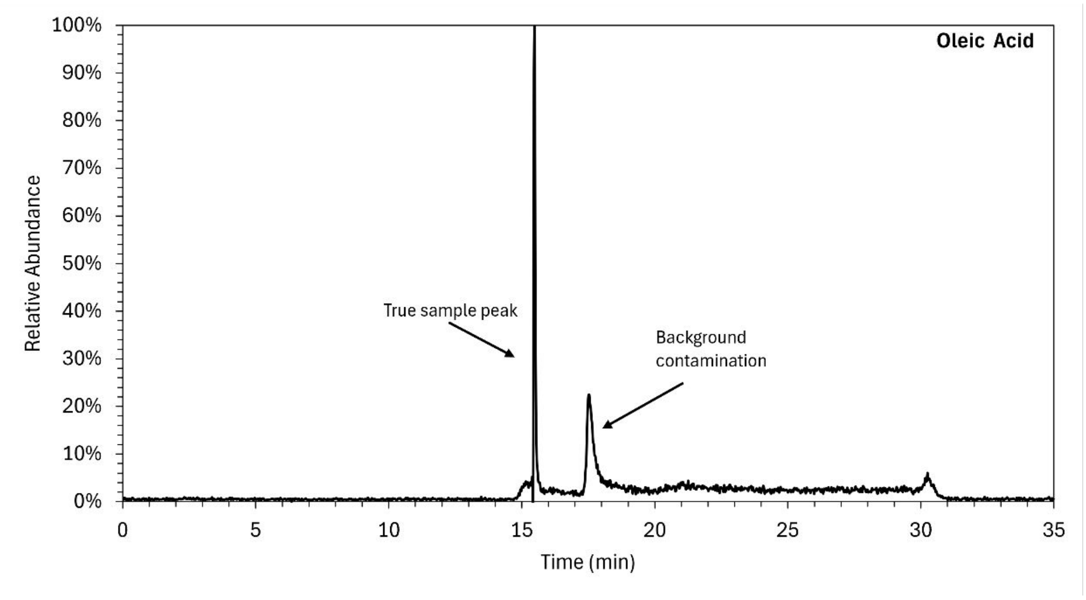

Figure S.4: Example extracted ion chromatogram for oleic acid in a standard sample corresponding to the m/z range of 281.2458-281.2514. The first large peak at RT 15.48 indicates the presence of the fatty acid in the injected sample, whilst the second peak at RT 17.54 shows the fatty acid that is present as contamination in the eluents.

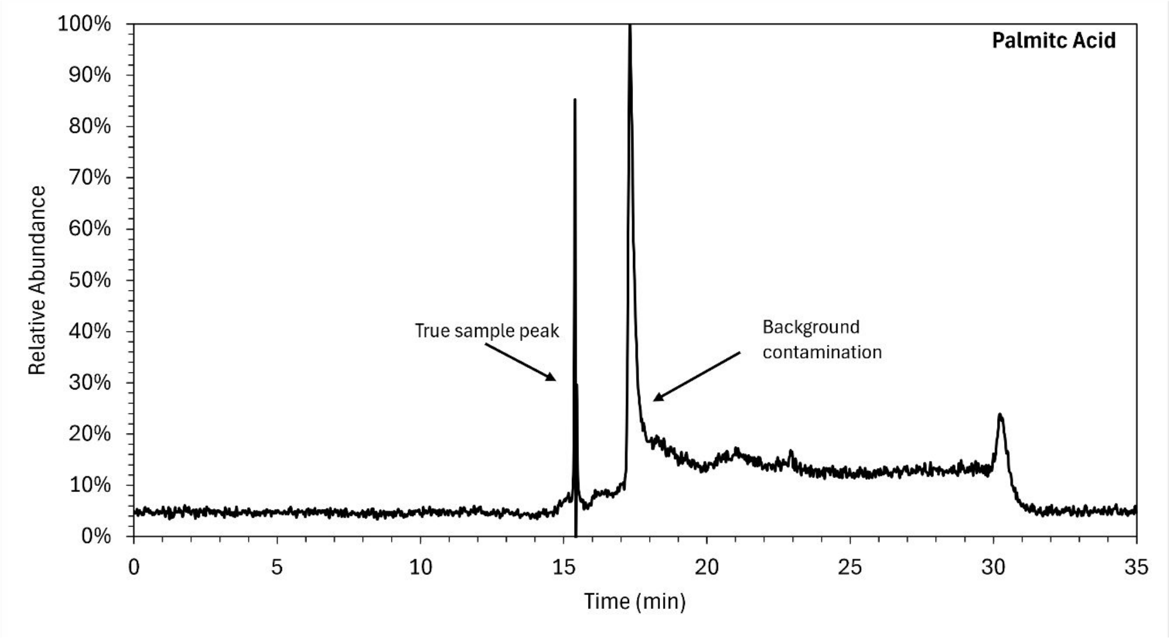

Figure S.5: Example extracted ion chromatogram for palmitic acid in a standard sample corresponding to the m/z range of 255.2304-255.2356. The first large peak at RT 15.40 indicates the presence of the fatty acid in the injected sample, whilst the second peak at RT 17.31 shows the fatty acid that is present as contamination in the eluents.

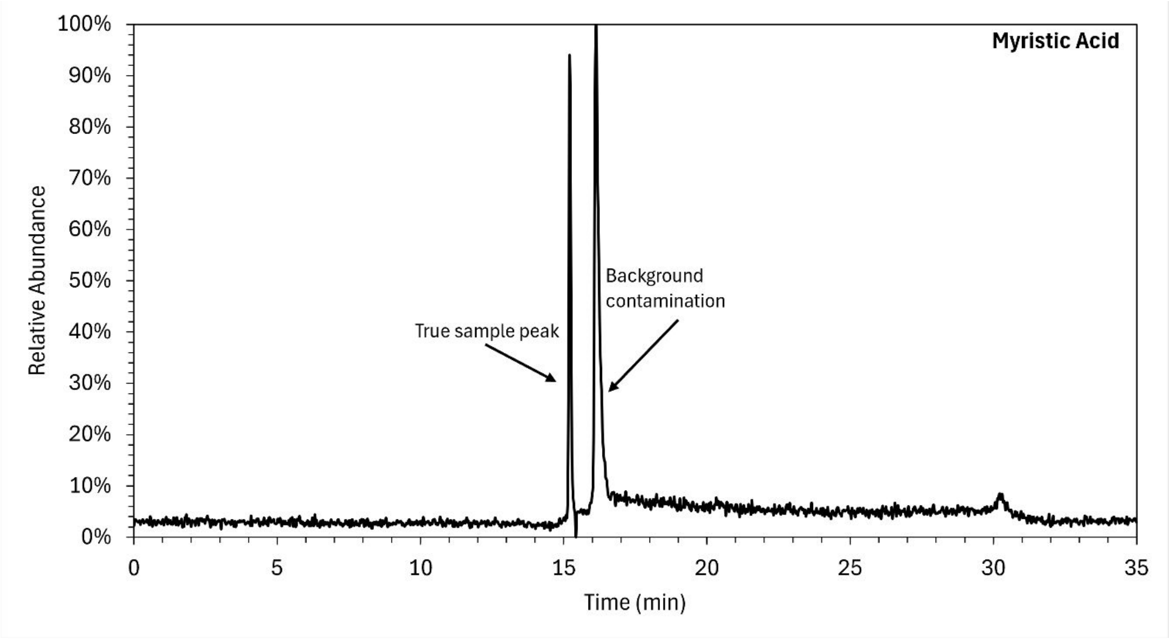

Figure S.6: Example extracted ion chromatogram for myristic acid in a standard sample corresponding to the m/z range of 227.1994-227.2040. The first large peak at RT 15.20 indicates the presence of the fatty acid in the injected sample, whilst the second peak at RT 16.13 shows the fatty acid that is present as contamination in the eluents.

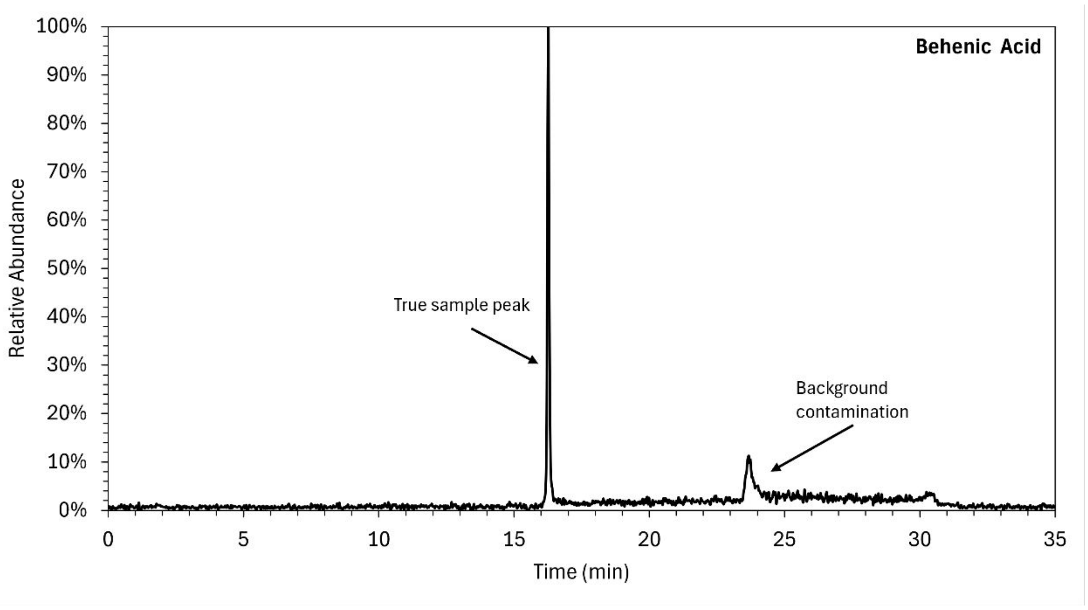

Figure S.7: Example extracted ion chromatogram for behenic acid in a standard sample corresponding to the m/z range of 339.3232-339.330. The first large peak at RT 16.24 indicates the presence of the fatty acid in the injected sample, whilst the second peak at RT 23.68 shows the fatty acid that is present as contamination in the eluents.

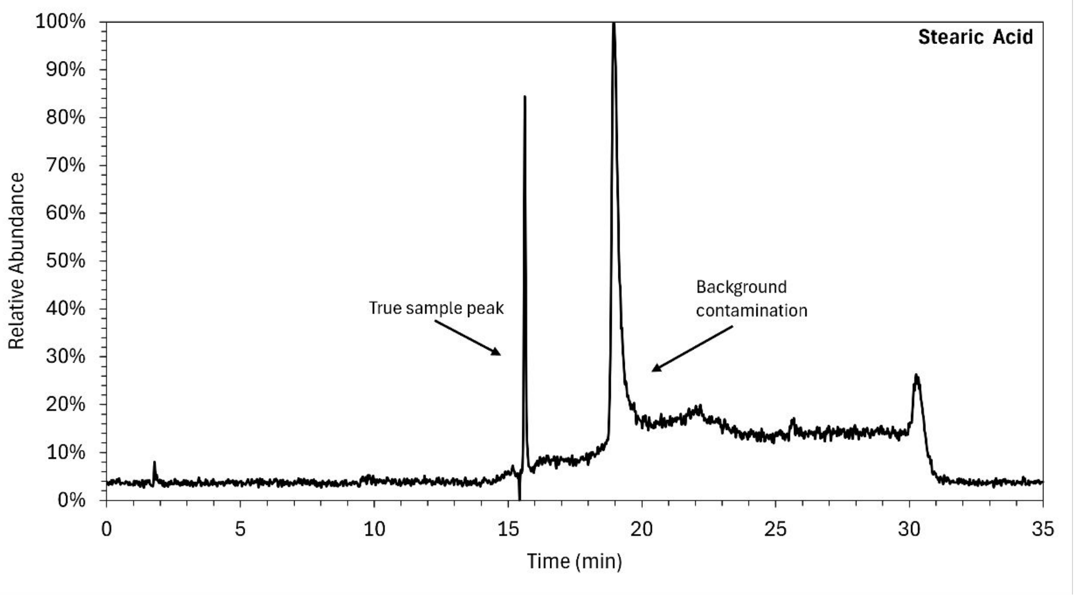

Figure S.8: Example extracted ion chromatogram for stearic acid in a standard sample corresponding to the m/z range of 283.2614-283.2670. The first large peak at RT 15.62 indicates the presence of the fatty acid in the injected sample, whilst the second peak at RT 18.94 shows the fatty acid that is present as contamination in the eluents.

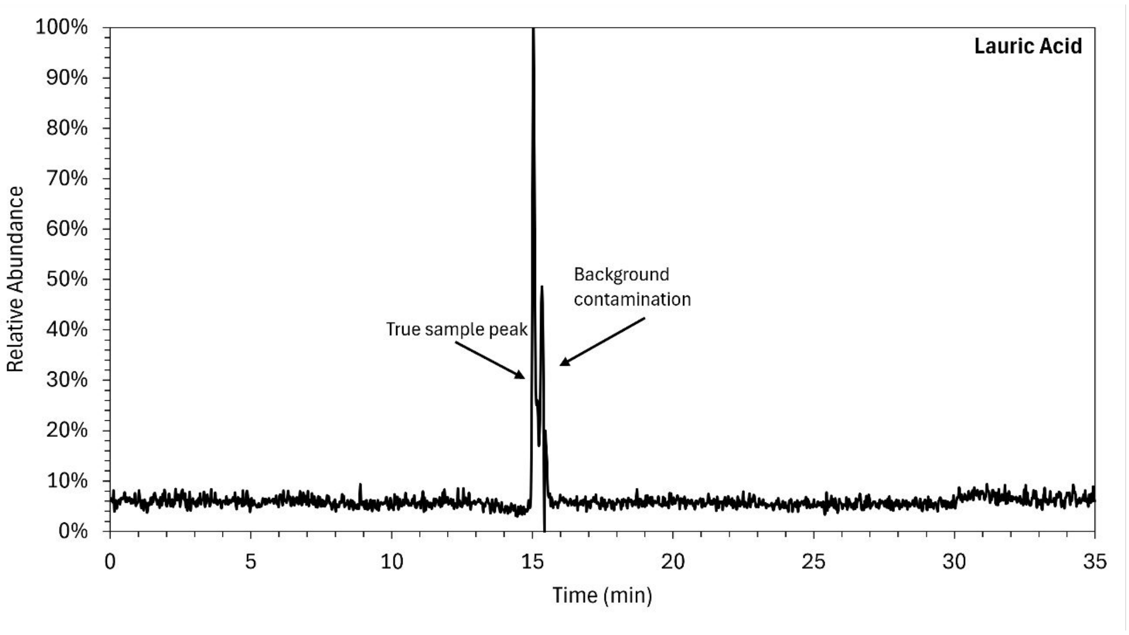

Figure S.9: Example extracted ion chromatogram for lauric acid in a standard sample corresponding to the m/z range of 199.1679-199.1719. The first large peak at RT 15.03 indicates the presence of the fatty acid in the injected sample, whilst the second peak at RT 15.34 shows the fatty acid that is present as contamination in the eluents.

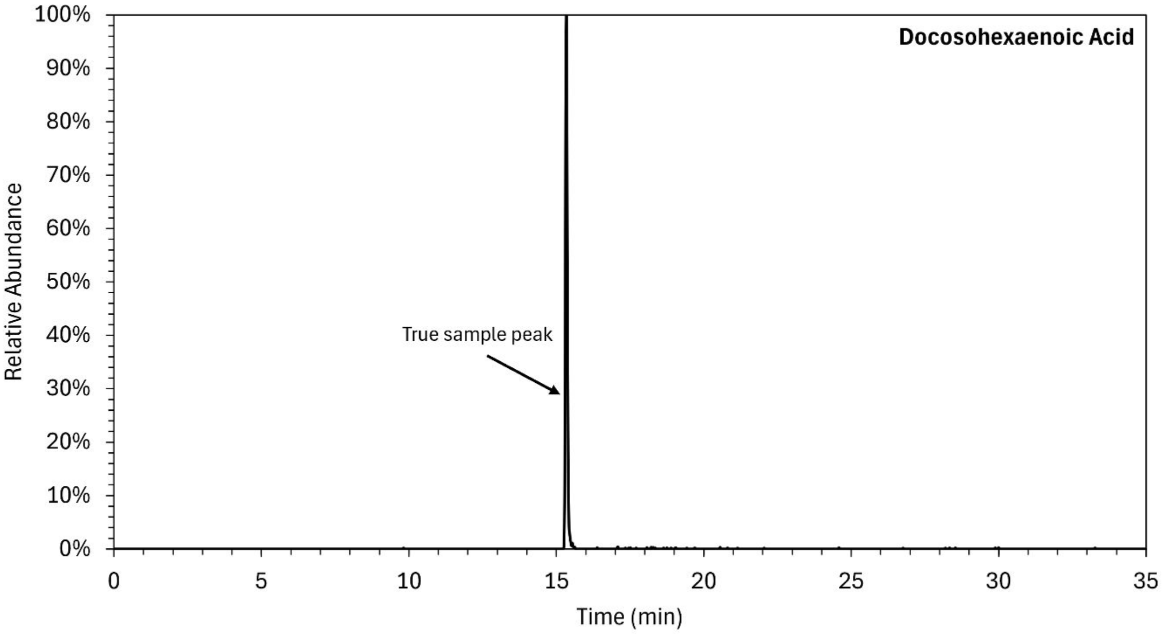

Figure S.9: Example extracted ion chromatogram for lauric acid in a standard sample corresponding to the m/z range of 199.1679-199.1719. The first large peak at RT 15.03 indicates the presence of the fatty acid in the injected sample, whilst the second peak at RT 15.34 shows the fatty acid that is present as contamination in the eluents.

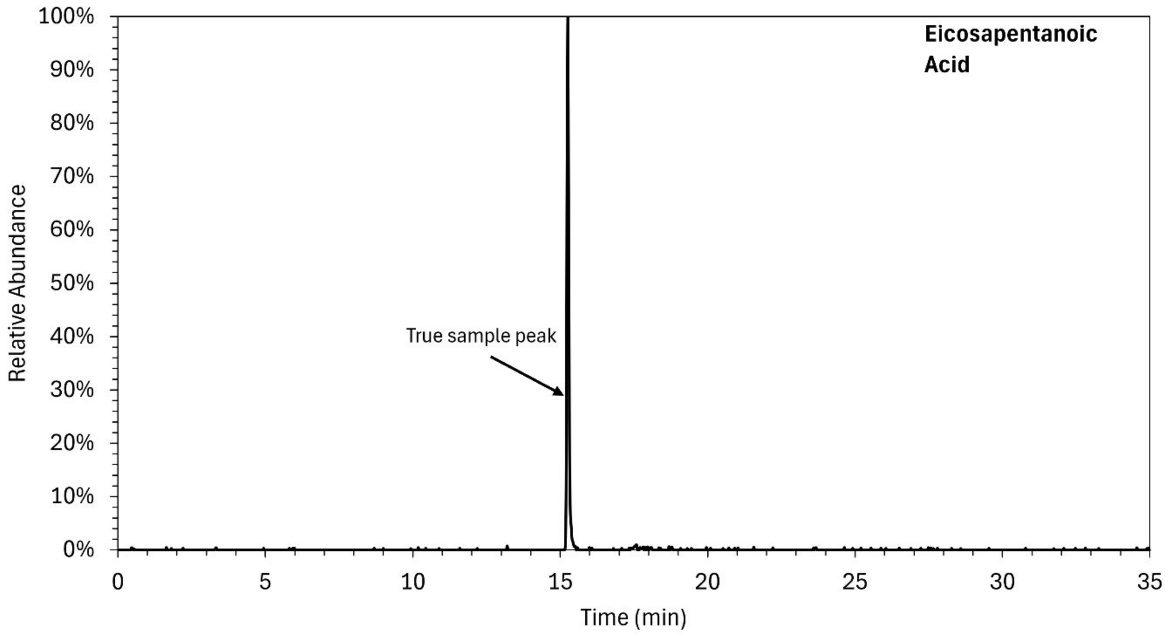

Figure S.11: Example extracted ion chromatogram for eicosapentaenoic acid in a standard sample corresponding to the m/z range of 301.2072-301.2272. The first large peak at RT 15.26 indicates the presence of the fatty acid in the injected sample.

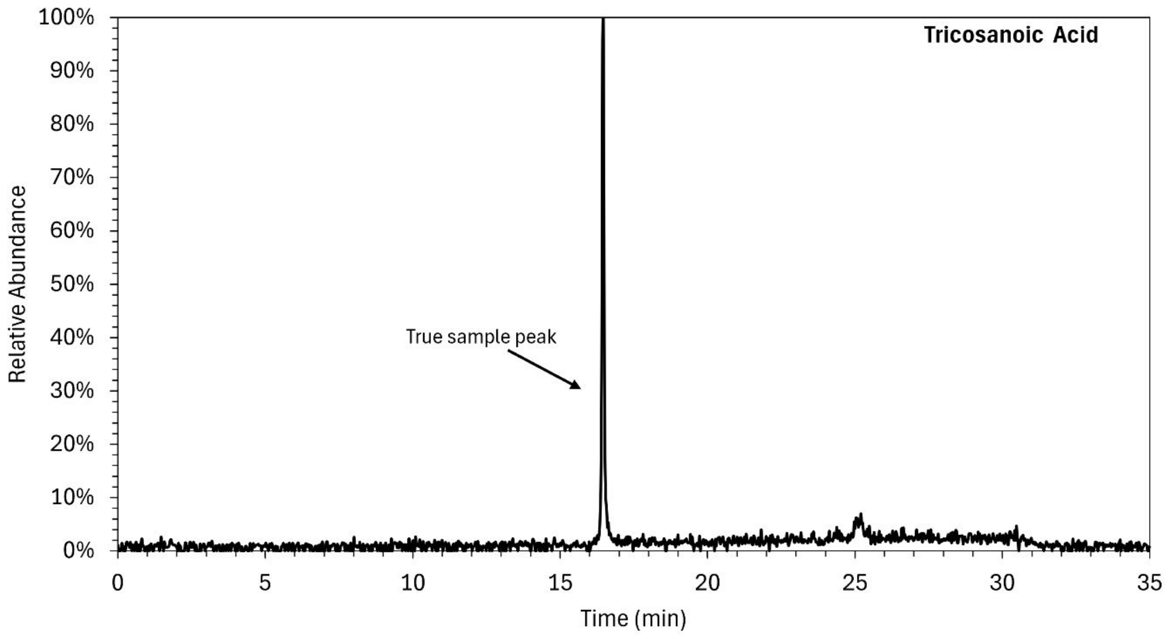

Figure S.12: Example extracted ion chromatogram for tricosanoic acid in a standard sample corresponding to the m/z range of 353.3325-353.3525. The first large peak at RT 16.47 indicates the presence of the fatty acid in the injected sample.

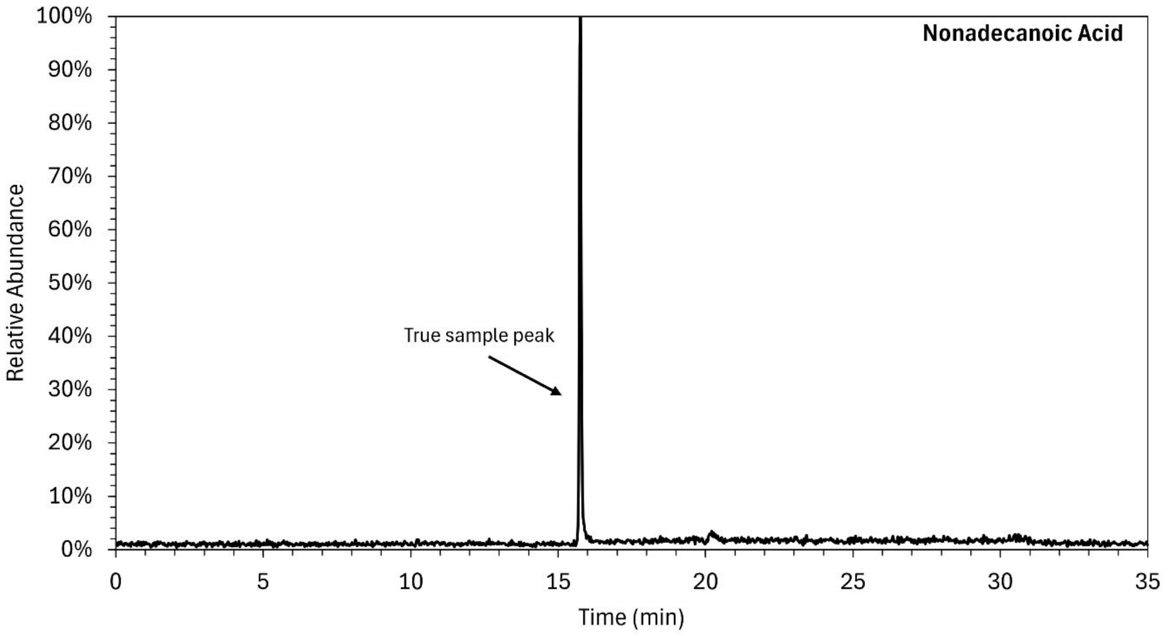

Figure S.13: Example extracted ion chromatogram for nonadecanoic acid in a standard sample corresponding to the m/z range of 297.2697-297.2897. The first large peak at RT 15.76 indicates the presence of the fatty acid in the injected sample.

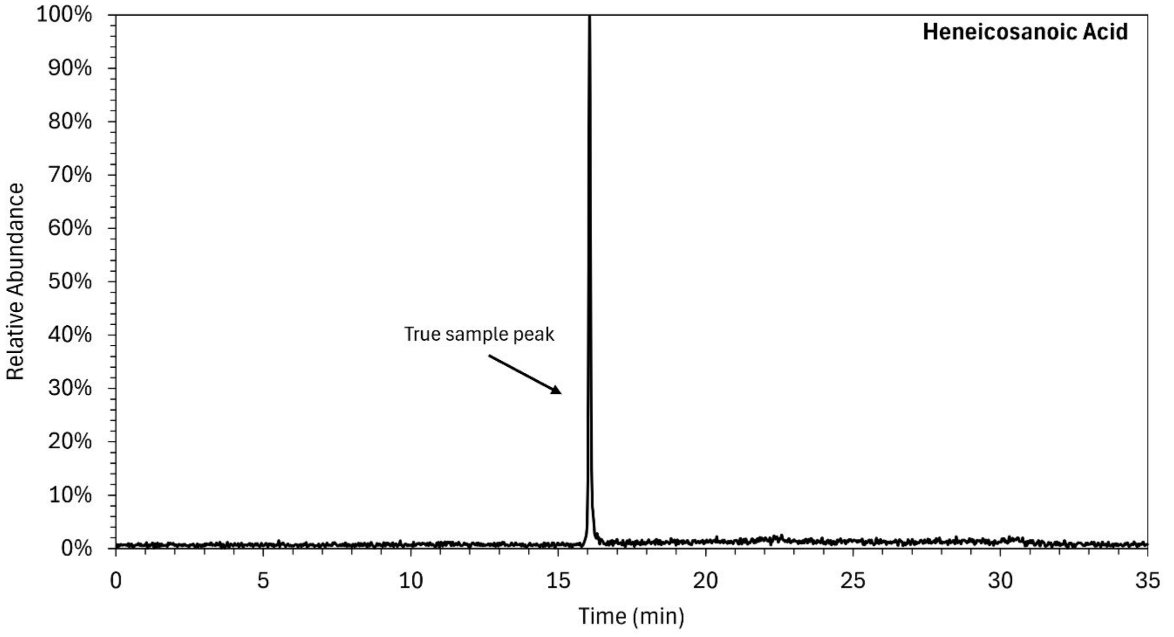

Figure S.14: Example extracted ion chromatogram for heneicosanoic acid in a standard sample corresponding to the m/z range of 325.3012-325.3212. The first large peak at RT 16.07 indicates the presence of the fatty acid in the injected sample.

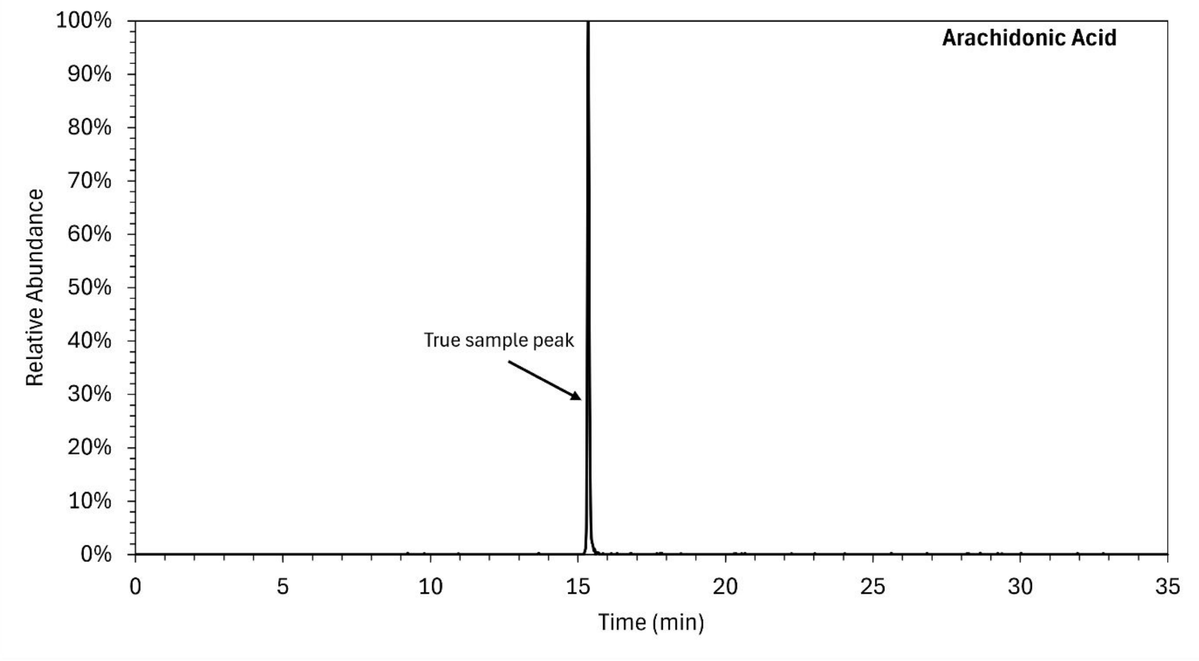

Figure S.15: Example extracted ion chromatogram for arachidonic acid in a standard sample corresponding to the m/z range of 303.2228-303.2428. The first large peak at RT 15.34 indicates the presence of the fatty acid in the injected sample.

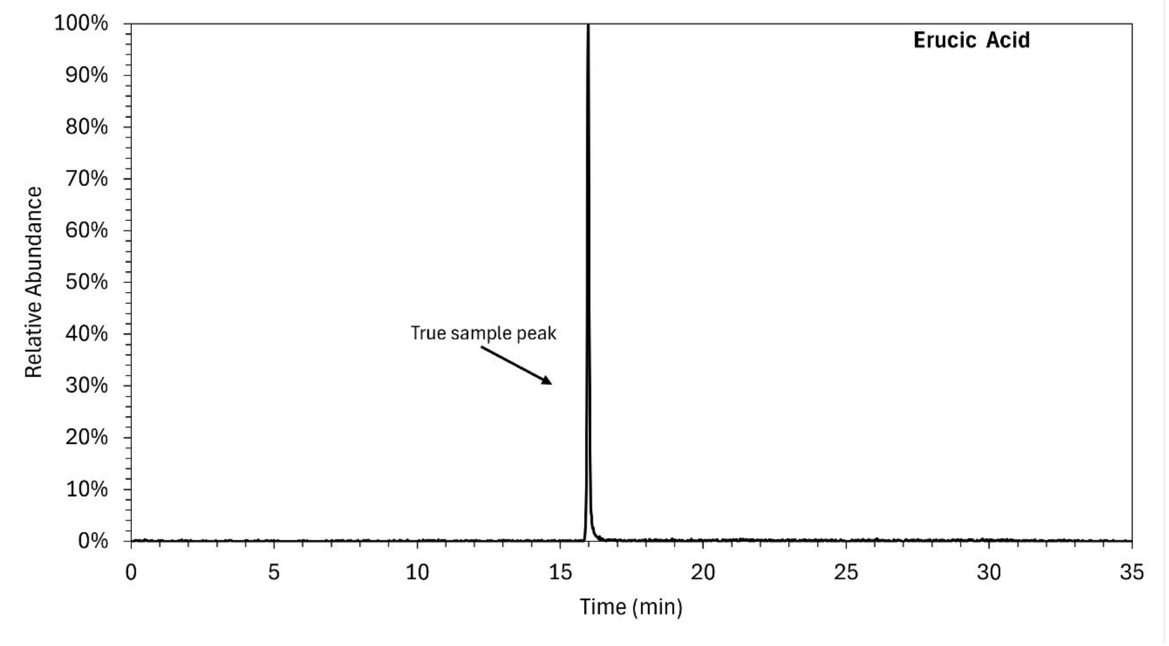

Figure S.16: Example extracted ion chromatogram for erucic acid in a standard sample corresponding to the m/z range of 337.3012-337.3212. The first large peak at RT 15.99 indicates the presence of the fatty acid in the injected sample.
